# Characterization of the Humoral Immune Response during *Staphylococcus aureus* Bacteremia and Global Gene Expression by *Staphylococcus aureus* in Human Blood

**DOI:** 10.1371/journal.pone.0053391

**Published:** 2013-01-07

**Authors:** Paul Martijn den Reijer, Nicole Lemmens-den Toom, Samantha Kant, Susan V. Snijders, Hélène Boelens, Mehri Tavakol, Nelianne J. Verkaik, Alex van Belkum, Henri A. Verbrugh, Willem J. B. van Wamel

**Affiliations:** Department of Medical Microbiology and Infectious Diseases, Erasmus University Medical Center Rotterdam, Rotterdam, The Netherlands; Rush University Medical Center, United States of America

## Abstract

Attempts to develop an efficient anti-staphylococcal vaccine in humans have so far been unsuccessful. Therefore, more knowledge of the antigens that are expressed by *Staphylococcus aureus* in human blood and induce an immune response in patients is required. In this study we further characterize the serial levels of IgG and IgA antibodies against 56 staphylococcal antigens in multiple serum samples of 21 patients with a *S. aureus* bacteremia, compare peak IgG levels between patients and 30 non-infected controls, and analyze the expression of 3626 genes by two genetically distinct isolates in human blood. The serum antibody levels were measured using a bead-based flow cytometry technique (xMAP®, Luminex corporation). Gene expression levels were analyzed using a microarray (BµG@s microarray). The initial levels and time taken to reach peak IgG and IgA antibody levels were heterogeneous in bacteremia patients. The antigen SA0688 was associated with the highest median initial-to-peak antibody fold-increase for IgG (5.05-fold) and the second highest increase for IgA (2.07-fold). Peak IgG levels against 27 antigens, including the antigen SA0688, were significantly elevated in bacteremia patients versus controls (*P*≤0.05). Expression of diverse genes, including *SA0688,* was ubiquitously high in both isolates at all time points during incubation in blood. However, only a limited number of genes were specifically up- or downregulated in both isolates when cultured in blood, compared to the start of incubation in blood or during incubation in BHI broth. In conclusion, most staphylococcal antigens tested in this study, including many known virulence factors, do not induce uniform increases in the antibody levels in bacteremia patients. In addition, the expression of these antigens by *S. aureus* is not significantly altered by incubation in human blood over time. One immunogenic and ubiquitously expressed antigen is the putative iron-regulated ABC transporter SA0688.

## Introduction

*Staphylococcus aureus* is one of the most common causes of bloodstream infections [1], [2] and *S. aureus* bloodstream infections are associated with serious complications such as infective endocarditis and prosthetic device infection [3], [4], [5]. The mortality rate of *S. aureus* bacteremia is approximately 20–30% [6], [7], [8]. Unfortunately, due to the increasing antibiotic resistance of clinical *S. aureus* isolates [9], [10] and the simultaneous decrease in the number of newly approved antimicrobial agents [11], [12], the treatment of *S. aureus* bacteremia is becoming increasingly difficult. Therefore, alternative strategies to prevent or treat *S. aureus* bacteremia are much needed.

One potential strategy is the development of a vaccine. However, despite the promising results of anti-staphylococcal vaccines in animal models, efforts to develop an efficient vaccine against *S. aureus* in humans have so far failed [13], [14], [15]. Classically, vaccine development has focused on stimulating the humoral immune response during *S. aureus* infection, as this response is considered to play an important role in clearing infections [16]. Although recent work questions the effectiveness of the humoral immune response in clearing infections [15] and suggests a more important role for the Th17 cell-mediated immune response [14], [17], knowledge of which antigens are expressed by bacteria and are immunogenic in infected patients remains essential for new immunotherapies. However, to date the number of reports exploring the immunogenicity of *S. aureus* antigens, especially in humans, is limited and all of these studies have investigated relatively small numbers of bacterial antigens (reviewed in [16]. In short, two of the most recent studies found detectable yet heterogeneous antibody levels in single serum samples of both infected patients and healthy controls against 19 [18] or 8 [19] recombinant *S. aureus* antigens. Another study analyzed the immunogenicity of whole-cell wall protein preparations using 2-dimensional gel electrophoresis (2-DE) immunoblotting of pooled sera from both infected patients and controls [20]. Fifteen immunogenic surface proteins were identified, including SdrE and SA0688, for which significantly increased IgG levels had previously been demonstrated in infected patients, compared to non-infected controls [18]. In the most comprehensive study to date, the antibody levels against 19 staphylococcal antigens were serially measured in multiple serum samples from bacteremia patients [21], and heterogeneity in the antibody levels between different patients was again observed. IsdA was associated with increased antibody levels in the majority of patients and was therefore suggested as a potential vaccine component. However, several antigens analyzed in earlier studies including wall teichoic acid, peptidoglycan, SA0688, alpha toxin and other antigens which are hypothesized to be important virulence factors were not analyzed in this study.

To further characterize the humoral immune response during *S. aureus* bacteremia, we analyzed the levels of IgG and IgA antibodies against 56 staphylococcal antigens in serial serum samples from 21 bacteremia patients. This is the largest collection of known staphylococcal antigens analyzed to date, including the non-protein antigens wall-teichoic acid and peptidoglycan. In addition, we compared the IgG levels against all 56 antigens in bacteremia patients and non-infected controls. Finally, to gain further insight into the bacterial antigens that are expressed in human blood and could be involved in the pathogenesis of bacteremia, we studied the expression changes of 3626 *S. aureus* genes during the incubation of two genetically distinct strains in human blood using microarray analyses. Based on these investigations and previous data, we discuss the potential of specific staphylococcal antigens as components of human vaccines.

## Materials and Methods

### Ethics Statement

All patient serum samples used in this study were obtained from coded left-over material from routine diagnostic blood samples. In concordance with the guidelines of the Erasmus University Medical Hospital and the Dutch federation of Biomedical Scientific Societies (Federatie van Medische Wetenschappelijke Verenigingen), all patients were informed of the possibility that left-over material from diagnostic samples could be used for scientific research and all patients were offered the opportunity to give written refusal to this. Serum samples used in this study were only obtained from patients who did not object to the use of left-over material for scientific research and gave verbal consent for this. This procedure was approved and the acquisition of additional written consent was waived specifically for this retrospective study by the Medical Ethics Committee of the Erasmus University Medical Center Rotterdam (MEC-2007-106, addendum 2). All collected serum samples were coded and only qualified physicians of the department of Medical Microbiology and Infectious Diseases had access to the original patient data.

### Patients, Controls and Definitions

Twenty-one adult patients, admitted to the Erasmus Medical Center between March 2007 and March 2011 were followed from the time of diagnosis of *S. aureus* bacteremia until discharge from the hospital or, if applicable, during outpatient appointments after discharge. Bacteremia was defined as the isolation of *S. aureus* from at least one blood culture set. A median number of 10 (interquartile range, 12) serum samples were collected per patient over a median period of 25 (interquartile range, 37) days. The median age of the bacteremia patients included in the study was 66 years (interquartile range, 10 years), of whom 71% were male.

All patients were treated with antibiotics according to hospital guidelines under the supervision of a consultant of the Department of Medical Microbiology and Infectious Diseases. During admission to the hospital, 4 of the 22 patients died; however, none of these deaths could be directly attributed to staphylococcal bacteremia.

Single serum samples were collected from 30 non-infected patients, admitted to the Erasmus Medical Center between July 2011 and February 2012 for reasons other than any infectious disease. All control patients did not suffer from any clinically apparent infection in at least the past 6 months. The median age of the non-infected control patients was 62 years (interquartile range, 11.5 years), of whom 80% were male. *S. aureus* nasal carrier status was not tested for either the bacteremia patients or the control group. However, previous results [22] and additional data (not shown) suggest that there is no overall significant difference in the IgG levels of persistent carriers and non-carriers for all of the antigens tested in this study, except for TSST-1 and SasG.

### *S aureus* Strains, Detection of Virulence Genes and Genotyping

*S. aureus* isolates from bacteremia patients were identified on the basis of colony and cellular morphology and Slidex Staph Plus agglutination testing (bioMérieux, Marcy l'Etoile, France). The identification of all staphylococcal isolates was confirmed by Staphylococcus protein A (*spa*)-PCR [23]. The obtained PCR fragments were sequenced; these sequences formed the basis of *spa*-typing. All of the isolates were methicillin-sensitive, as determined by the cefoxitin disk diffusion test according to the Clinical and Laboratory Standards Institute (CLSI) criteria [24]. Antimicrobial susceptibility to additional antibiotics was determined using the VITEK® 2 system with card AST-P549 (bioMérieux).

For each of the 21 bacteremia patients, the first *S. aureus* isolate obtained from a blood culture was screened using PCR for the presence of the 54 genes encoding the antigens to which the antibody responses were measured. PCR was not performed for peptidoglycan and wall teichoic acid biosynthesis genes, as these were assumed to be obligatorily present in all isolates. Primers were both newly designed (Table 1) or described previously [21], [25]. In addition, pulsed-field gel electrophoresis (PFGE) was performed on *Sma1-*digested chromosomal DNA from all 21 isolates, as described previously [26]. Relatedness among the PFGE profiles was evaluated using Bionumerics software (version 3.0; Applied Maths, Ghent, Belgium).

**Table 1:** Newly designed primers used in this study.

Gene	Forward	Reverse
Alpha toxin	CGGGATCCGCAGATTCTGATATTAATATT	AACTGCAGTTAATTTGTCATTTCTTCTT
*EsxA*	CTTACGGGCAAGGTTCAGAC	CTTGTTCTTGAACGGCATCA
*EsxB*	GGGTGGATATAAAGGTATTAAAGCA	ATGGGTTCACCCTATCAAGC
*ETA*	ACTGTAGGAGCTAGTGCATTTGT	TGGATACTTTTGTCTATCTTTTTCATCAAC
*ETB*	ACAAGCAAAAGAATACAGCG	GTTTTTGGCTGCTTCTCTTG
*FlipR*	TCGCTGCAGGTCTTTTAACTC	GCTTTCTTCACATCACCTTGG
*HlgB*	GTCAGAGAGTCCATAATGCATTTAA	CACCAAATGTATAGCCTAAAGTG
*IsaA*	ACCTGAAGCACCTGATGGGT	TACGCAGCAGGTACAGGACA
*Lipase*	CAATAGGCGTGGTGTCAGTG	AATCGCCAACTTGTGGTTTC
*LukDE*	TGAAAAAGGTTCAAAGTTGATACGAG	TGTATTCGATAGCAAAAGCAGTGCA
*LukF*	ATCATTAGGTAAAATGTCTGGACATGATCCA	GCATCAA(GC)TGTATTGGATAGCAAAAGC
*LukS*	GCAGACGCGTCAACACAA	TTTTACATTTTCCCTATCTTTTT
*LytM*	CATGCGAAAGACGCAAGCTG	AGGCGCTGTTGAATTACCCG
*Nuc*	TTATTAAGTGCTGGCATATGTATG	TTTTCTAATATTAAATACACTTAC
*PrsA*	AAGCAATACGGCGGTAAAGA	GTGCGCCACCTTGTTTAAGT
*SSL1*	TTCAATTTTTGCATTTTGAGGTT	TTCTTCATCTGAAGCGAAAGC
*SSL3*	TCGAGTATGACTTCAATTTGTGC	GAACCACATCAACACAACTTCC
*SSL5*	GATGACAGCAATTGCGAAAG	ATAGCCGCCATCTTTCATTG
*SSL9*	ATCGGCCAATGCAGAAGTAG	CCACCGACCGAGTATTTGTC
*SSL10*	CAGCATTAGCAAAAGCGACA	GCTTTCTATGACTTCCCCCATA
*SSL11*	GCACTAGGGATTTTAACAACAGG	CCATGCGATGAGGCTGTAAT
*SEC*	CTTGTATGTATGGAGGAATAACAA	TGCAGGCATCATATCATACCA
*SED*	GTGGTGAAATAGATAGGACTGC	ATATGAAGGTGCTCTGTGG
*SEE*	TACCAATTAACTTGTGGATAGAC	CTCTTTGCACCTTACCGC
*SEG*	CGTCTCCACCTGTTGAAGG	CCAAGTGATTGTCTATTGTCG
*SEH*	CAACTGCTGATTTAGCTCAG	GTCGAATGAGTAATCTCTAGG
*SEN*	CGTGGCAATTAGACGAGTC	GATTGATTTGATGATTATAG

### Bacterial Antigens

All *S. aureus* antigens used for Luminex experiments were 6× His-tagged recombinant proteins with the exception of the synthetic phenol-soluble modulin α 1–4 peptides and the sugars petidoglycan and wall-teichoic acid. The following antigens were coupled to xMAP® beads (Luminex Corporation, Austin, TX, USA): protein secretion system ESX-1-associated factors EsxA and B; Nuclease (Nuc); peptidoglycan hydrolase LytM; immunodominant antigen A (IsaA); glucosaminidase; lipase; peptidoglycan (PG); wall teichoic acid (WTA); foldase-protein PrsA; clumping factor A and B (ClfA and ClfB); SD-repeat containing proteins D and E (SdrD and SdrE); iron-responsive surface determinants A and H (IsdA and IsdH); fibronectin-binding proteins A and B (FnbpA and FnbpB); extracellular fibrinogen-binding protein (Efb); *S. aureus* surface protein G (SasG); staphylococcal complement inhibitor (SCIN); chemotaxis inhibitory protein of *Staphylococcus aureus* (CHIPS); formyl peptide receptor-like inhibitory protein (FLIPr); phenol-soluble modulin α 1–4 peptides (PSM alpha 1–4), alpha toxin; gamma-hemolysin B (HlgB); leukocidins D, E, F and S (LukD, LukE, LukF and LukS); staphylococcal enterotoxins A–E, G–J, M–O, Q, R (SEA–SEE, SEG-SEJ, SEM-SEO, SEQ, SER); exfoliative toxins A and B (ETA and ETB); toxic shock syndrome toxin 1 (TSST-1); staphylococcal superantigen-like proteins 1, 3, 5, 9, 10 and 11 (SSL1, SSL3, SSL5, SSL9, SSL10 and SSL11) and hypothetical proteins SA0104, SA0486 and SA0688. The following purified non-staphylococcal proteins were also coupled to xMAP beads as negative controls: *Moraxella catarrhalis* ubiquitous surface protein 1 (UspA1); *Streptococcus pneumoniae* pneumococcal surface adhesin A (PsaA) and human metapneumovirus surface protein (hMPV).

SasG, SdrD, SdrE, ClfB, IsdA, IsdH, FnbpA and FnbpB were expressed and purified as described previously [27]. The constructs were kindly provided by T. Foster (Trinity College, Dublin, Ireland). Alpha toxin, HlgB, LukD, LukE, LukF, LukS, SEA and SEC were prepared as described previously [28]. All other proteins were kindly provided by other research groups, as indicated in the acknowledgments.

The purity of all proteins was confirmed using SDS-page. The antigens were coupled to xMAP® beads as described previously [29], [30] with some modifications for PG, WTA and PSM alpha 1–4. For PG and WTA, the beads were incubated with the cross-linkers adipic acid dihydrazide (ADH; 35 mg/ml) and EDC (200 mg/ml) before incubation with the antigens according to the standard protocol. For PSM alpha 1–4 peptides, the activated beads were firstly coupled to 25 µg streptavidin per reaction according to the standard protocol, and then subsequently coupled to biotin-labeled PSM alpha 1–4 peptides for one hour.

### Measurement of Anti-staphylococcal Antibodies

The levels of IgG and IgA antibodies against 56 staphylococcal antigens in the serum samples of bacteremia patients were measured using a bead-based flow cytometry technique (xMAP®; Luminex Corporation), as previously described [21], [29], [30]. In addition to staphylococcal antigens, the IgG levels against the non-staphylococcal proteins UspA1 (*Moraxella cattharalis*), PsaA (*Streptococcus pneumoniae*) and hMPV (human metapneumovirus) were also determined. Serum samples were diluted 1∶100 in PBS and secondary phycoerythrin (PE)-labeled goat anti-human antibodies against either total IgG or IgA were diluted 1∶200. All measurements were performed in duplicate and the median fluorescence intensities (MFIs), a semi-quantitative measure of antibody levels, were averaged. Duplicate measurements for which the coefficient of variation was larger than 25% were excluded from further analysis. All measurements were corrected for non-specific background signal by subtracting the MFIs of control beads not coupled to any protein.

For the determination of immunological cross-reactivity, 1∶200 diluted serum from one bacteremia patient with high MFIs for all leukocidins was pre-incubated with recombinant proteins serially diluted in PBS for 35 minutes on a thermomixer plate shaker. After incubation the serum was spun down twice for 10 minutes at 3400 RPM and non-bound specific antibodies remaining in the supernatant were measured following the standard protocol.

### Microarray Experiments

Two *S. aureus* isolates from different bacteremia patients were used for the microarray experiments. Overnight cultures were diluted 100 times in fresh prewarmed brain-heart infusion (BHI) broth and grown at 37°C in 5% CO_2_ until an OD_590_ of 0.5 was reached. A volume of 30 ml of the culture was pelleted, and then resuspended in 5 ml of freshly isolated heparinized human blood or BHI broth, and incubated with gentle rotation at 37°C in 5% CO_2_. All experiments were independently repeated twice with blood from two healthy volunteers. Both volunteers were persistent nasal carriers of *S. aureus*, which was confirmed with 3 positive nasal swabs taken with a regular interval of 7 days over a period of 3 weeks. At time point 0 minutes for BHI and time point 0, 30, 60 and 90 minutes for blood, 10 ml RNA protect (Qiagen, Germantown, MD, USA) was added to the samples and incubated for 5 minutes at roomtemperature. The cultures were then pelleted, cold water was added and subsequently 10 x concentrated PBS was added. After centrifugation the pellets were lysed using 1 ml RLT buffer (Qiagen) and 10 µl β-mercaptoethanol, and finally the bacterial pellets were resuspended in 1 ml RNA Pro solution (Qbiogene Inc., City, CA, USA).

*S. aureus* RNA was isolated using the FastRNA® Pro Blue Kit according to the manufacturer’s instructions (Qbiogene Inc.) using the Fastprep FP120 instrument (Qbiogene; two cycles of 45 seconds at a speed setting of 6.0). After isolation, the RNA was treated with 6 U TURBO DNase (Ambion, Austin, TX, USA) according to the manufacturer’s instructions, and then the RNA was further purified using the RNAeasy kit (Qiagen) following the manufacturer’s protocol.

Chromosomal DNA was isolated from overnight cultures grown in BHI broth. Bacteria were lysed using FastProtein™ Blue Matrix and the FastPrep® instrument (Qbiogene; two cycles of 45 seconds at a speed setting of 6.0). DNA was then purified using the QIAamp DNA Mini Kit (Qiagen) and treated with 10 µl RNase (Promega, Madison, WI, USA).

Hybridization probes were generated from 5 µg total RNA or 1 µg DNA according to the protocol of the Bacterial Microarray Group (BµG@s; St. George’s Hospital Medical School, London, UK). RNA or DNA was mixed with 3 µg random primers (Invitrogen, Breda, The Netherlands), heat denatured and snap cooled on ice. The RNA was reverse transcribed to cDNA to incorporate the Cy5 dCTP (GE Healthcare, Diegem, Belgium) fluorescent analog, and DNA was labeled with Cy3 dCTP (GE Healthcare). Labeled RNA and DNA samples were pooled, and hybridized overnight to an *S. aureus* microarray with PCR amplicons printed on Ultragaps (Corning, NY, USA) glass slides (BµG@S) [31]. The array design is available in BµG@Sbase (Accession No. A-BUGS-17; http://bugs.sgul.ac.uk/A-BUGS-17) and also ArrayExpress (Accession No. A-BUGS-17).

The microarray slides were scanned using the ScanArray Express HT scanner (Perkin Elmer, Groningen, The Netherlands) following the manufacturer’s instructions. The spots were quantified using Imagene 6.0 software (BioDiscovery, Marina Del Ray, CA, USA). The fully annotated microarray data have been deposited in BµG@Sbase (accession number E-BUGS-137; http://bugs.sgul.ac.uk/E-BUGS-137) (http://bugs.sgul.ac.uk/E-BUGS-137%29) and also ArrayExpress (accession number E-BUGS-137). GeneSpring GX version 7.3 Software (Agilent Technologies, Santa Clara, CA, USA) was used for normalization and further data analysis. Expression levels were quantified as the log ratio of the signal derived from RNA isolated from blood divided by the signal derived from DNA isolated from broth. Expression levels were averaged for the duplicate experiments from each blood donor, and then the average expression levels from both donors were averaged.

### Statistical Analysis

Fold-increases in antibody levels were calculated as the ratio of the peak antibody level divided by the initial antibody level (as measured in the first serum sample). If the antibody level only declined after the initial measurement, than the ratio of the lowest antibody level divided by the initial antibody level was calculated. Both fold-increases and decreases were pooled to determine the median fold-change in antibody levels per antigen.

Evaluation of histogram plots and the Kolmogorov-Smirnov test revealed a non-normal distribution of the IgG levels for most antigens. The non-parametric Mann-Whitney U test was used to compare the antibody levels of bacteremia patients and controls. Spearman’s correlation coefficient was used for correlation analysis of the microarray data. *P*-values ≤0.05 were considered statistically significant. All statistical analyses were performed using SPSS version 15.0 (SPSS, Chicago, IL, USA) or Graphpad Prism version 5 (Graphpad Inc. La Jolla, CA, USA).

## Results

### Genetic Typing and Presence of Virulence Genes in Clinical *S. aureus* Isolates

**Figure 1:**
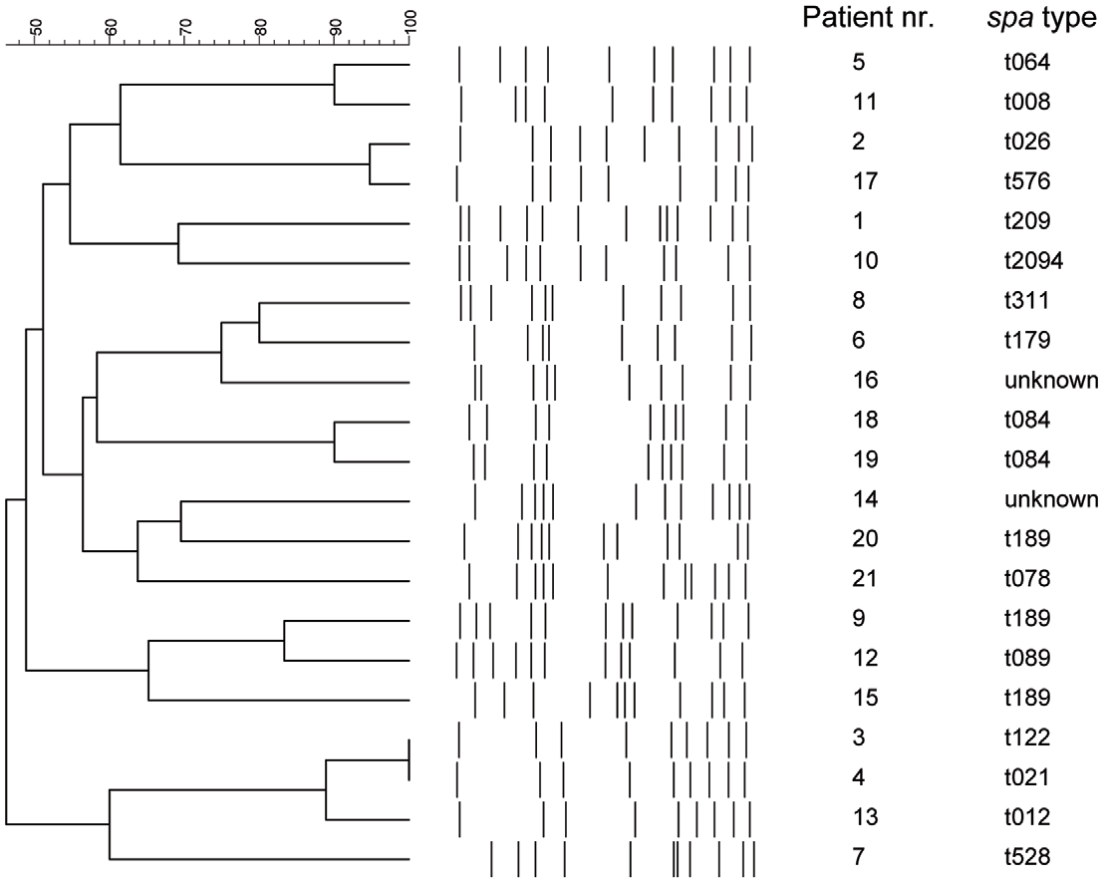
Dendogram of clinical isolates. Pulsed-field gel electrophoresis data and *spa*-types of *S. aureus* isolates obtained from blood cultures of 21 bacteremia patients are shown.

PFGE analysis was performed on the first available *S. aureus* isolates from all 21 bacteremia patients. The dendogram in Figure 1 illustrates the overall lack of relatedness between the isolates from different patients, with the exception of the isolates from patients 4, 5 and 14. However, there was no epidemiological relationship between these or any of the patients included in this study. To further characterize the genetic background of the clinical isolates, all strains were *spa*-typed. A broad range of *spa*-types linked to different clonal clusters were observed, including two unknown new *spa*-types. All of the isolates were methicillin-sensitive.

For 54 of the 56 antigens analyzed in this study, the presence of the corresponding genes was determined in all clinical isolates using PCR. In addition to the biosynthesis genes for peptidoglycan and wall teichoic acid which are obligatorily present in each isolate, 11 genes were found to be ubiquitously present in all isolates: alpha toxin, clumping factor A and B, glucosaminidase, *IsaA*, *IsdA*, lipase, *LytM*, nuclease, *PrsA* and *SA0688*. Five genes were present in only one isolate: exfoliative toxin A, leukocidins F and S, and staphylococcal enterotoxins C and Q. Exfoliative toxin B and enterotoxins E and H were not present in any of the isolates. A summary of the number of isolates containing each gene is presented in Table 2.

**Table 2:** Overview of gene presence and associated IgG responses of bacterial antigens.

Antigen	No. of patients with gene pos isolates (%)	No. of patients with increase in IgG level (%)	Median fold increase frominitial to peak level (range)	Significant difference	p value
Alpha toxin	21/21 (100%)	19/21 (91%)	1.3 (0.9–3.46)	ND	ND
CHIPS	13/21 (62%)	15/17 (88%)	1.2 (0.4–2.95)	no	0.271
ClfA	21/21 (100%)	17/19 (90%)	1.6 (0.02–6.09)	yes	0.010
ClfB	21/21 (100%)	13/19 (68%)	1.1 (0.01–5.42)	yes	0.036
Efb	20/21 (95%)	10/14 (71%)	1.6 (0.51–7.96)	no	0.492
EsxA	21/21 (100%)	CV>25%	CV>25%	ND	ND
EsxB	14/21 (67%)	14/16 (88%)	1.4 (0.22–2.91)	yes	0.022
ETA	1/21 (5%)	14/21 (67%)	1.4 (0.29–23.78)	no	0.246
ETB	0/21 (0%)	14/20 (70%)	1.30 (0.29–23.78)	no	0.738
FlipR	15/21 (72%)	17/20 (85%)	1.32 (0.43–8.02)	yes	0.005
FnbpA	19/21 (91%)	13/14 (93%)	1.41 (0.04–5.15)	no	0.181
FnbpB	6/21 (29%)	8/9 (89%)	1.39 (0.75–3.68)	yes	0.052
Glucosaminidase	21/21 (100%)	20/20 (100%)	1.38 (1.02–25.75)	yes	<0.0001
HlgB	16/21 (76%)	21/21 (100%)	1.26 (1.02–5.14)	yes	0.001
IsaA	21/21 (100%)	19/20 (95%)	1.09 (0.9–13.2)	yes	0.003
IsdA	21/21 (100%)	21/21 (100%)	1.74 (1.07–40.45)	yes	0.005
IsdH	20/21 (95%)	15/17 (88%)	2.0 (0.62–6.6)	yes	0.011
Lipase	21/21 (100%)	20/21 (95%)	1.34 (0.85–20.99)	yes	0.002
LukD	15/21 (71%)	21/21 (100%)	1.37 (0.64–4.63)	yes	0.008
LukE	15/21 (71%)	20/21 (95%)	1.3 (0.64–4.63)	yes	0.004
LukF	0/21 (0%)	20/21 (95%)	1.54 (0.95–3.92)	yes	0.0002
LukS	0/21 (0%)	21/21 (100%)	1.37 (1.02–4.99)	yes	0.003
LytM	21/21 (100%)	18/21 (86%)	1.33 (0.25–32.08)	no	0.768
Nuc	21/21 (100%)	20/20 (100%)	1.55 (1.0–9.48)	yes	0.025
Peptidoglycan	ND	18/20 (90%)	1.26 (0.3–4.53)	yes	0.009
PrsA	21/21 (100%)	12/12 (100%)	2.92 (1.32–34.21)	no	0.096
PSMa peptides 1–4	21/21 (100%)	CV>25%	CV>25%	ND	ND
SA0104	16/21 (76%)	CV>25%	CV>25%	ND	ND
SA0486	17/21 (81%)	7/9 (78%)	1.37 (0.46–3.43)	yes	0.020
SA0688	21/21 (100%)	18/19 (95%)	5.17 (0.74–56.96)	yes	<0.0001
SasG	11/21 (52%)	7/10 (70%)	1.17 (0.35–21.64)	no	0.374
SCIN	20/21 (95%)	19/20 (95%)	1.39 (0.92–16.58)	yes	0.0005
SdrD	17/21 (81%)	11/12 (92%)	1.37 (0.01–5.17)	yes	0.049
SdrE	14/21 (67%)	16/18 (89%)	1.69 (0.7–14.59)	no	0.405
SEA	2/21 (14%)	16/21 (76%)	1.16 (0.3–52.14)	no	0.486
SEB	5/21 (24%)	6/8 (75%)	1.13 (0.28–5.21)	no	0.203
SEC	1/21 (5%)	18/21 (86%)	1.11 (0.68–19.37)	no	0.356
SED	2/21 (10%)	18/21 (86%)	1.39 (0.56–8.54)	no	0.130
SEE	0/21 (0%)	15/20 (76%)	1.39 (0.51–7.29)	no	0.327
SEG	11/21 (52%)	15/21 (71%)	1.13 (0.11–5.07)	no	0.106
SEH	0/21 (0%)	13/21 (62%)	1.11 (0.08–3.8)	no	0.329
SEI	11/21 (52%)	CV>25%	CV>25%	ND	ND
SEJ	2/21 (10%)	CV>25%	CV>25%	ND	ND
SEM	8/21 (38%)	8/12 (67%)	1.54 (0.12–7.79)	no	0.080
SEN	10/21 (48%)	17/21 (81%)	1.34 (0.62–4.72)	no	0.053
SEO	11/21 (52%)	10/14 (71%)	1.08 (0.098–2.03)	no	0.111
SEQ	1/21 (5%)	5/10 (50%)	1.05 (0.03–1.42)	no	0.334
SER	2/21 (10%)	14/20 (70%)	1.44 (0.46–10.09)	no	0.298
SSL1	18/21 (86%)	17/21 (81%)	1.56 (0.7–11.21)	yes	0.001
SSL3	19/21 (91%)	20/21 (95%)	1.29 (0.9–3.87)	yes	0.002
SSL5	21/21 (100%)	19/21 (90%)	1.90 (0.65–8.43)	yes	<0.0001
SSL9	6/21 (29%)	17/21 (81%)	1.35 (0.77–10.15)	yes	0.003
SSL10	14/21 (67%)	18/19 (95%)	1.42 (0.84–4.83)	yes	0.046
SSL11	7/21 (33%)	19/21 (91%)	1.70 (0.67–8.67)	yes	0.015
TSST1	3/21 (14%)	16/21 (76%)	1.34 (0.27–10.69)	no	0.271
Wall teichoic acid	ND	17/19 (89%)	1.12 (0.29–6.26)	no	0.344
UspA1	ND	11/14 (79%)	1.21 (0.72–2.07)	ND	ND
PsaA	ND	10/12 (83%)	1.32 (0.7–2.07)	ND	ND
hMPV	ND	12/13 (92%)	1.11 (0.89–1.79)	ND	ND

Presence of genes in 21 isolates, initial-to-peak fold-increases in IgG levels and comparison of peak IgG levels in 21 bacteremia patients and 30 non-infected controls for 56 staphylococcal antigens and three non-staphylococcal control antigens. Patients for whom the duplicate measurements of the IgG levels had a CV larger than 25% were excluded from the analysis. IgG levels for the antigens EsxA, PSM alpha 1–4 peptides, SA0104, SEI and SEJ were completely excluded because of very low signal intensities with coefficients of variation larger than 25% for a majority of patients. ND: not determined.

### Anti-staphylococcal Antibodies in Bacteremia Patients

To study the humoral immune response against a wide array of staphylococcal antigens in bacteremia patients, the total IgG and IgA levels against 56 antigens were measured in serial serum samples from 21 bacteremia patients. The IgG and IgA levels against EsxA, PSM alpha 1–4 peptides, SA0104, SEI and SEJ, and additionally IgA levels against EsxB and SEO were excluded from further analysis, due to the very low signal intensities with coefficients of variation larger than 25% between duplicate experiments.

**Figure 2:**
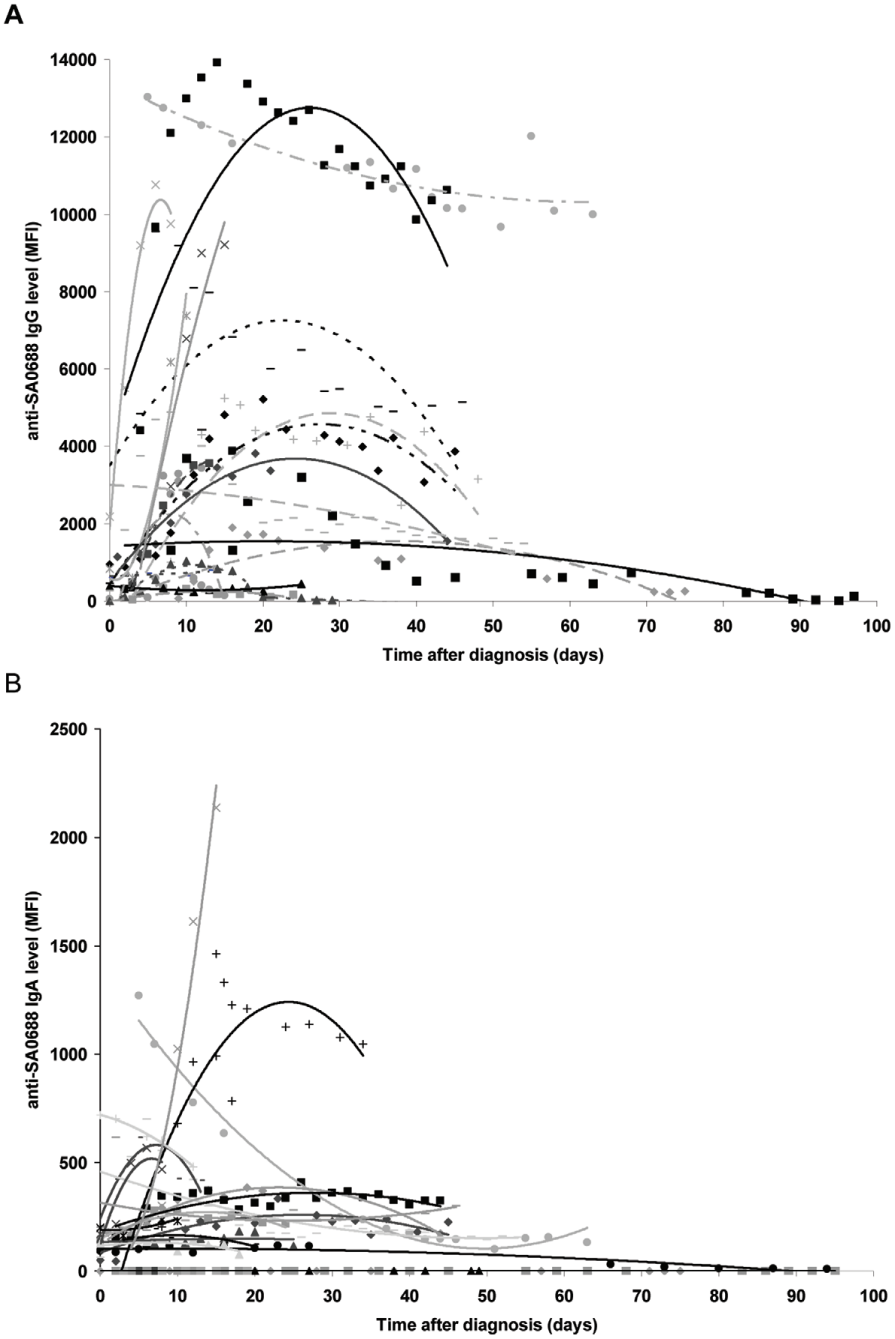
Antibody courses against the antigen SA0688 in bacteremia patients. A: Courses of anti-SA0688 IgG levels in 21 bacteremia patients. Each data point represents the average of duplicate Luminex measurements expressed as the mean fluorescence intensity (MFI). Each patient is represented by a serial set of the same symbols and a polynomial trend lines is drawn through this set. B: Course of anti-SA0688 IgA levels in 21 bacteremia patients. Note that the MFI values on the Y-axis are a factor of 10 lower than in Figure 2A.

In general, IgG levels directed against all antigens were already detectable at the time of diagnosis and showed a temporal increase in the majority of bacteremia patients. The increases in IgA levels were generally many-fold lower than the increases in IgG levels. The levels of antigen-specific IgG, and to a lesser extent IgA, varied extensively in the first serum sample obtained from each patient (median of 0 days after diagnosis; range, 0–5 days) (Figure 2). The time taken to reach the peak antibody levels varied widely between patients and antigens, and ranged from 7 to 86 days after diagnosis. The course of antibody levels after reaching the peak height was generally characterized by a decrease back towards the initial level and remaining at this level for up to 97 days after diagnosis (Figure 2).

For 15 antigens, an increase in IgG levels was observed at some time point after the onset of bacteremia in 95 to 100% of all patients: glucosaminidase, HlgB, IsaA, IsdA, lipase, leukocidins D, E, S and F, nuclease, PrsA, SA0688, SCIN and SSL3 and 10 (Table 2). In contrast to these 15 antigens, only PrsA and Efb were associated with increased IgA levels in 95 to 100% of all patients (Table S1). However, due to the lower signal intensities and coefficients of variation larger than 25% between duplicate experiments, PrsA and Efb-associated IgA levels in the majority of patients were excluded from further analysis. No other antigens were associated with increased IgA and/or IgG levels in at least 95% of all patients.

**Figure 3:**
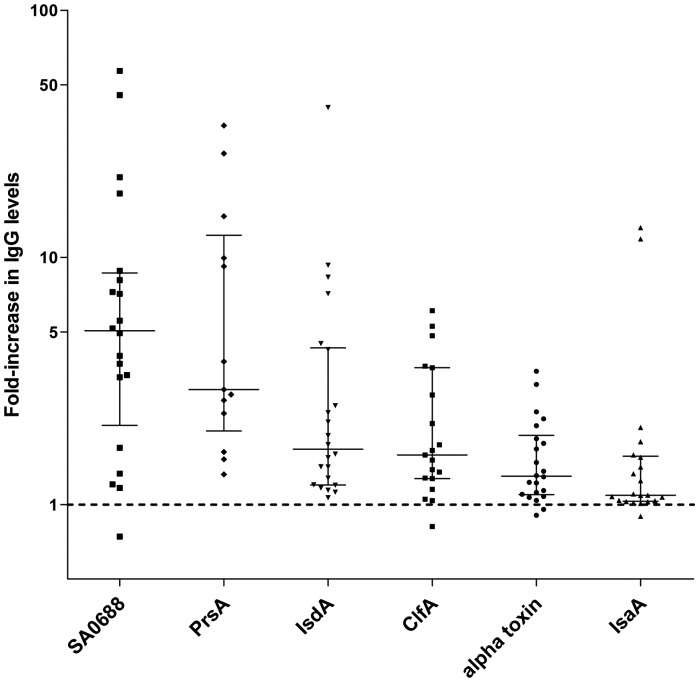
Initial-to-peak fold-increases in IgG levels for 6 bacterial antigens in bacteremia patients. Each data point represents a single patient; the median fold-increase in IgG levels and interquartile range are represented by lines. Patients for whom the duplicate Luminex measurements had a CV >25% were excluded from the analysis. Note the log10 scale of the y-axis.

When the increases in the IgG levels against each bacterial antigen in individual patients were combined, the highest median fold increase from the initial serum sample to the peak IgG level was observed for SA0688 (5.17-fold increase; range, 0.74–56.96) followed by PrsA (2.92-fold increase; range, 1.32–34.21; Figure 3, Table 2). PrsA was also associated with the highest median fold increase in IgA levels (3.92-fold increase, range, 1.02–20.83), followed by SasG (2.51-fold increase; range, 0.63–13.02) and SA0688 (2.07-fold increase; range, 0.67–11.31) (Table S2). All other antigens showed median fold increases close to the overall median increase of 1.33 for IgG and 1.42 for IgA.

The median fold increases in the IgG levels for the non-staphylococcal control antigens UspA1, PsaA and hMPV were 1.21 (range 0.72–2.07), 1.32 (range 0.7–2.07) and 1.11 (range 0.89–1.79), respectively.

For 289 (35%) of the 832 observed increases in IgG levels, the corresponding gene was not present in the *S. aureus* isolate from the same patient as determined by PCR. Of these 289 ‘false-positive’ increases, 259 (90%) were observed for known excreted antigens, mainly exfoliative toxins, enterotoxins and hemolysins. When all of the initial-to-peak fold-increases were classified as occurring in either the presence or absence of the corresponding genes in respective isolates, an overall median fold increase of 1.37 (range, 0.85–18.07) was observed in the presence of corresponding genes and 1.29 (range, 0.83–2.44) in the absence of corresponding genes.

### Comparison of Anti-staphylococcal Antibodies in Patients and Controls

**Figure 4:**
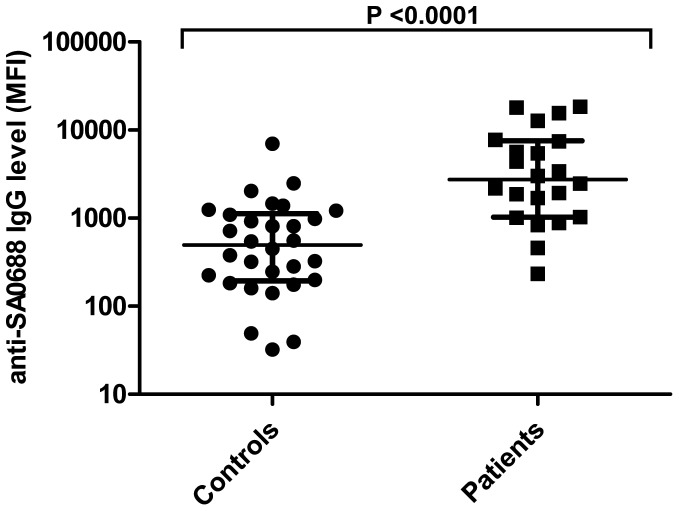
Comparison of anti-SA0688 IgG levels in bacteremia patients and non-infected controls. Peak IgG levels of 21 bacteremia patients were compared to IgG levels of 30 non-infected controls. The median value and interquartile range are represented by lines. Note the log10 scale of the y-axis.

To investigate the significance of the increased IgG levels observed in bacteremia patients, the peak IgG levels of all 21 bacteremia patients were compared to the IgG levels of 30 non-infected, age-matched control patients. The IgG levels directed against 27 antigens were significantly higher in bacteremia patients than the non-infected controls (Table 2). The antigens associated with the most significant elevations in IgG levels in bacteremia patients compared to controls were SA0688 (Figure 4) and glucosaminidase (*P*<0.0001), the immune modulators SSL5 (*P*<0.0001), SSL1 (*P*  = 0.0007) and SCIN (*P*  = 0.0004), and the toxins gamma-hemolysin B (*P*  = 0.0007) and leukocidin F (*P*<0.0001).

### In vitro Expression of Bacterial Antigens in Human Blood

**Figure 5:**
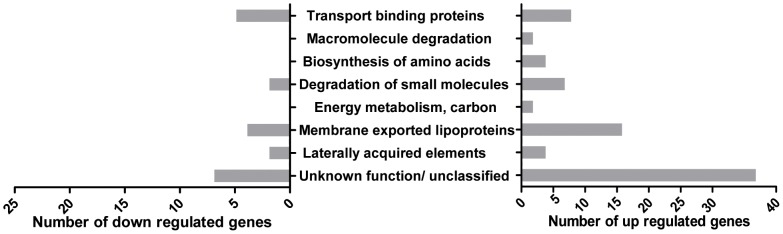
Functional distribution of genes with altered mRNA expression in human blood. The functional classes are shown for which the largest number of genes showed an at least twofold increased or decreased mRNA expression at all timepoints in blood (30, 60 and 90 minutes) in both strains compared to trancriptomes at the start of incubation in blood (0 minutes). Functional classes for which only one gene showed significant alterations in mRNA expression in blood are not shown.

To gain further insight into which bacterial antigens are expressed in human blood and could be involved in the pathogenesis of bacteremia, microarray experiments were performed using the genetically distinct isolates from patients 1 and 3 (Figure 1) to measure the global changes in *S. aureus* mRNA expression during culture in human blood. The mRNA expression levels of 3626 *S. aureus* genes were measured during log-phase growth in BHI broth and also after 0, 30, 60 and 90 minutes culture in human blood. Compared to the transcriptomes at the start of incubation in blood (0 minutes), only 86 out of the 3626 tested genes showed a two-fold or higher increase in mRNA expression in both isolates at all time points (30, 60 and 90 minutes) when incubated in blood (Table S2). A majority of these upregulated genes have an unknown/unclassified function, are involved in carbon metabolism or are excreted lipoproteins (Figure 5). The only known virulence factors for which mRNA expression was upregulated after culture in blood were the IgG-binding protein *sbi* and the gamma-hemolysin A and B precursors. Thirty genes showed a two-fold or more reduction in mRNA expression in both isolates at all time points, compared to the transcriptomes of both isolates at the start of incubation in blood (Table S2). These downregulated genes are also mainly involved in cellular metabolism or have an unknown function (Figure 5). Comparison of the transcriptomes at each individual time point (30, 60 and 90 minutes) with the transcriptomes of both isolates at the start of incubation in blood (0 minutes), revealed that a total of 360, 420 and 641 genes, respectively, were up- or downregulated two-fold or more. The functional distribution of the differentially expressed genes at each time point was similar to the functional distribution of the differentially expressed genes at all time points combined. In addition to the earlier mentioned upregulation of hemolysin precursors, a more than two-fold upregulation of *IsdA, -B, -C and –F, FnbpA* and *B* and *ClfA* was noted after 90 minutes incubation in blood; whereas only *IsdA, -B, -C, -D* and *FnbpA* were upregulated two-fold or higher after 60 minutes and only *IsdC* was upregulated at least two-fold after 30 minutes, compared to the transcriptomes of both isolates at the start of incubation in blood.

Compared to log-phase growth in BHI broth, only 7 of the 3626 analyzed genes showed a two-fold or higher increase in mRNA expression in both strains at all time points (30, 60 and 90 minutes) when cultured in blood: dihydrolipoamide succinyltransferase, the sugar phospate antiporter *uhpT*, the murein hydrolase regulatory gene *IrgA* and the transcripts encoding the putative proteins SA0806, 0211, 0622 and 0761.

Of the 56 bacterial antigens for which the antibody responses were characterized in bacteremia patients, microarray data for 35 genes was available for at least two time points per isolate. In general, the mRNA expression levels of these 35 antigens, quantified as the RNA:DNA log ratios, correlated significantly between both isolates at all time points (*P*≤0.001), indicating similar expression levels for these specific 35 genes in both strains. The mRNA expression levels of four genes were consistently high in both isolates during log-phase growth in BHI broth and during all measured time points (0, 30, 60 and 90 minutes) of culture in blood: *SA0688, IsaA, EsxA* and *SCIN* (Table S3). In addition, the mRNA expression level of *PrsA* was high at all time points, except for the 90 minutes time point in one isolate. Compared to the expression level during log-phase growth in BHI broth, none of these 35 genes displayed a two-fold or higher mRNA expression level at any time point during culture in blood.

## Discussion

In this study we investigated the humoral immune response against 56 staphylococcal antigens in bacteremia patients. Firstly, we further demonstrate considerable variation in the IgA and IgG levels of all patients at the time of diagnosis; the time taken to reach peak antibody levels for each antigen in each patient was also heterogeneous. These heterogeneous, highly individual antibody responses are in line with previous data [18], [19], [21], [32] and will likely be the result of an individually unique interplay between patients and genetically diverse *S. aureus* strains. Indeed, parallel to the diversity of patient antibody responses we further confirmed the presence of a large genetic diversity amongst the infecting strains (figure 1).

The increases observed in the IgG levels of bacteremia patients were generally many-fold higher than the increases in the IgA levels. This may be explained either by hypothesizing that IgA production is not induced by hematogenic bacterial challenge to the same extent as IgG production, or that IgA levels may not alter considerably in blood but may increase more locally on mucosal surfaces. In any case, the relatively low IgA responses prompted us to focus attention on the more dynamic IgG responses in bacteremia patients.

The IgG levels against fifteen bacterial antigens, including well-described virulence factors such as IsdA and gamma-hemolysin B, were found to increase in at least 95% of the bacteremia patients. Additionally, the peak IgG levels against these 15 antigens were significantly higher in bacteremia patients than age-matched, non-infected patients. The putative ABC transporter SA0688 and the membrane-associated foldase PrsA were associated with the highest median fold increase in IgG levels (5.17 and 2.92-fold, respectively). Although other antigens were also associated with significantly increased IgG levels in individual patients, these data indicate that SA0688 and PrsA appear to be among the most broadly expressed and immunologically recognized antigens. This observation is in line with previous studies which demonstrated the immunogenicity of SA0688 in human serum [18], [20]. In addition, the antigen SA0688 showed promising results as part of a multivalent vaccine in an animal model of osteomyelitis [25]. Unfortunately, nothing is currently known about the exact function of SA0688 and how antibodies could interfere with staphylococcal infection by binding this antigen. Moreover, in general other antigens than SA0688 or PrsA which were not associated with significantly increased antibody levels in this study may also provide interesting targets for a vaccine, although we can only speculate about these antigens based on our data and previous studies.

In addition to the question which antigens should ideally be selected for a vaccine component, we can only speculate about whether or not the associated antibody responses will be protective against infection. We observed clearly detectable, pre-existent IgG levels against all antigens in patients at the time of diagnosis, which is in line with previous observations of stable, pre-existent IgG levels in both bacteremia patients and healthy controls [21], [27], [32]. These observations suggest that all individuals have an immunological memory specifically against *S. aureus*, possibly due to earlier, (sub)clinical infections. It remains a question whether a further increase in these pre-existent IgG levels will have an additional protective effect against infection, even though this increase is significant for diverse antigens such as SA0688 and PrsA compared to non-infected controls. In any case, the significant increases in IgG levels against diverse antigens suggests that these antigens are being expressed *in vivo* in patients, which will be a pre-requisite for any potential vaccine target.

To gain further insight into which bacterial antigens are expressed in human blood, the global changes in the mRNA expression levels of two genetically distinct *S. aureus* isolates during incubation in human blood were investigated. In general, of the 3626 genes investigated, we could only associate limited numbers of genes with significantly altered mRNA expression levels specifically during incubation in blood, compared to the transcriptomes of each isolate at the start of incubation in blood or BHI broth. As noted for 35 of the 56 antigens investigated in this study, most of the corresponding genes had a relatively constant RNA:DNA log ratio at all time points during culture in blood (Table S3). Most notably, the antigens *SA0688, IsaA, EsxA, SCIN* and, with the exception of one measurement, *PrsA* were highly expressed in both isolates in BHI broth and blood over time. This stable expression of genes by genetically distinct isolates in human blood or tissue would be a first prerequisite for any antigen to be a potential vaccine component.

Most of the genes which were up- or downregulated in *S. aureus* specifically during incubation in blood belong to functional classes involved in cellular metabolism or have an unknown function. Exceptions to this were the IgG-binding protein *sbi* and gamma-hemolysin component A precursor, which were upregulated in both isolates at all time points (30, 60 and 90 minutes) in blood compared to the start (0 minutes). In addition, other genes were upregulated at specific time points, mainly surface proteins such as *FnbpA, ClfA* and the diverse iron-regulated surface determinant (*Isd*) proteins. These findings are in agreement with a previous study which reported that a limited number of *S. aureus* genes encoding known virulence factors were specifically upregulated in blood [33]. In this study, mRNA expression of the gamma-hemolysin subunits were found to be most significantly upregulated during incubation in blood.

Although our study demonstrates the *in vitro* expression and *in vivo* immunogenicity of several antigens, there are several limitations in regard to the used techniques. Firstly, in regard to the bead-based flow cytometry assay, we used recombinant staphylococcal antigens in our assay which may lack certain naturally-occurring antibody-binding epitopes or may not have been optimally coupled to our assay beads, thereby possibly missing increases in the levels of specific antibodies. This could provide an alternative explanation for the low signal intensities observed for EsxA, PSM alpha 1–4 peptides, SA0104, SEI and SEJ. Secondly, we observed significant increases in the antibody levels against leukocidins S and F, for which corresponding genes were not present in any isolate. The increases in specific IgG for these two leukocidin components may be the result of immunological cross-reactivity, where antibodies specific to one toxin component may cross-react with structurally similar components [34], [35], [36]. We confirmed the presence of immunological cross-reactivity between the leukocidins F and D and gamma-hemolysin B in our assay (Figure S1). No cross-reactivity was observed between the enterotoxins in our assay (data not shown).

We observed a median fold-increase in IgG levels of 1.29 for all ‘false-positive’ increases where the corresponding genes were absent. This median ‘false-positive’ increase was comparable to that of all cases where corresponding genes were present (1.37) and the overall median fold-increase of 1.33. In addition, these increases were also comparable to those of the non-Staphylococcal control antigens UspA1, PsaA and hMPV (1.21, 1.32 and 1.11 fold-increase, respectively). This apparent background signal could on one hand be explained by a broad, non-specific rise in antibody levels during infection or, alternatively, by the statistical phenomenon that the maximum value of an extended time course will always be higher than single measurements. Indeed, although IgG levels tend to remain constant over time in both healthy persons [27] and up to three years after infection (unpublished data), small variations in measured IgG levels are consequently observed. This could explain the observed non-specific rise in antibody levels.

In regard to the micro array data, one limitation is that we investigated antigen expression in just two out of 21 strains. Investigating antigen expression in more strains would allow for more robust conclusions about global changes in bacterial transcriptomes, however we feel that we were able to gain more insight into the expression of specific genes with our current, rather technically demanding micro-array experiments. Secondly, as with other *in vitro* models the question remains how well our blood infection model reflects the *in vivo* situation during a bacteremia. Especially the high dose of bacteria used is likely different from the *in vivo* situation and could influence bacterial mRNA expression. Finally, in regard to the expression of antigens for which we characterized antibody responses, it should be noted that any direct correlation of *in vitro* bacterial gene expression with the *in vivo* immune response in patients should be interpreted with caution. The mode and phase of bacterial growth *in vivo* may be different and more diverse than the pattern of growth *in vitro*. In addition, the expression of a bacterial antigen does not necessarily induce an antibody response *in vivo*, either due to immune modulation by the bacterium or the complex regulatory immune processes within the host. Nonetheless, data on both bacterial gene expression *in vitro* and the *in vivo* immune response can yield valuable insight into the pathogenesis of infection and complement each other for the identification of potential vaccine targets.

To summarize, our study suggests that most of the staphylococcal antigens tested, including many known virulence factors, do not lead to uniform increases in the antibody levels in bacteremia patients. In addition, the expression of these antigens by *S. aureus* is not significantly altered by incubation in human blood over time. One immunogenic antigen is the putative iron-regulated ABC transporter SA0688, which induced a significant antibody response in all bacteremia patients and was stably expressed by genetically distinct isolates under different culture conditions. The ubiquitous expression of this antigen will be a prerequisite for any potential vaccine target and our data, together with previous literature, suggest that SA0688 could be a potential vaccine target.

## Supporting Information

Figure S1 **Cross-reactivity between leukocidins F and D and hemolysin gamma-B in human serum.** A: Serial dilutions of recombinant leukocidin F (LukF) were pre-incubated with the serum from a non-infected control with high IgG levels against LukF. After incubation, the remaining IgG levels specific against Leukocidins D, E, F and S and Hemolysin gamma-B were measured. Note the loss in IgG levels specific for LukD and HlgB at lower dilutions of LukF, suggesting immunological cross-talk between these toxin components. B: The same experiment as for Figure S1 A, now with serum from a different non-infected control.
(TIF)

Table S1 **Overview of gene presence and associated IgA responses of bacterial antigens.** Presence of genes in 21 isolates and initial-to-peak fold-increases in IgA levels in 21 bacteremia patients for 56 staphylococcal antigens. Patients for whom the duplicate measurements of the IgA levels had a CV larger than 25% were excluded from the analysis. IgA levels for the antigens EsxA, EsxB, PSM alpha 1–4 peptides, SA0104, SEI, SEJ and SEO were completely excluded because of very low signal intensities with coefficients of variation larger than 25% for a majority of patients. ND: not determined.
(DOC)

Table S2 **List of genes with altered mRNA expression in human blood.** Genes are listed for which mRNA expression is respectively at least twofold increased or decreased in both isolates during all time points (30, 60 and 90 minutes) of culture in blood compared to the transcriptomes at the start of culture in blood (0 minutes). mRNA expression is quantified as the average RNA:DNA log ratio of duplo experiments in separate blood samples of two blood donors. Ranges of RNA:DNA log ratios between duplo experiments in separate blood samples are given, unless only a single measurement from one blood sample was available.
(DOC)

Table S3 **mRNA expression levels of 35 genes in two isolates during culture in human blood and log-phase growth in BHI broth.** Average RNA:DNA log ratios of duplicate experiments in two separate blood samples are given; dark (red) cells indicate a RNA:DNA ratio larger than 2 (i.e. high expression) and gray (blue) cell indicates a RNA:DNA ratio smaller than 0.5 (i.e. low expression). Range of RNA:DNA log ratios between duplo experiments in seperate blood samples are given, unless only a single measurement from one blood sample was available.
(DOC)
