# Supplementary figures and images for: Characterization of the Humoral Immune Response during Staphylococcus aureus Bacteremia and Global Gene Expression by Staphylococcus aureus in Human Blood
Source: PLoS One. 2013 Jan 7;8(1):e53391. doi: 10.1371/journal.pone.0053391 (PMC3538780; doi:10.1371/journal.pone.0053391)

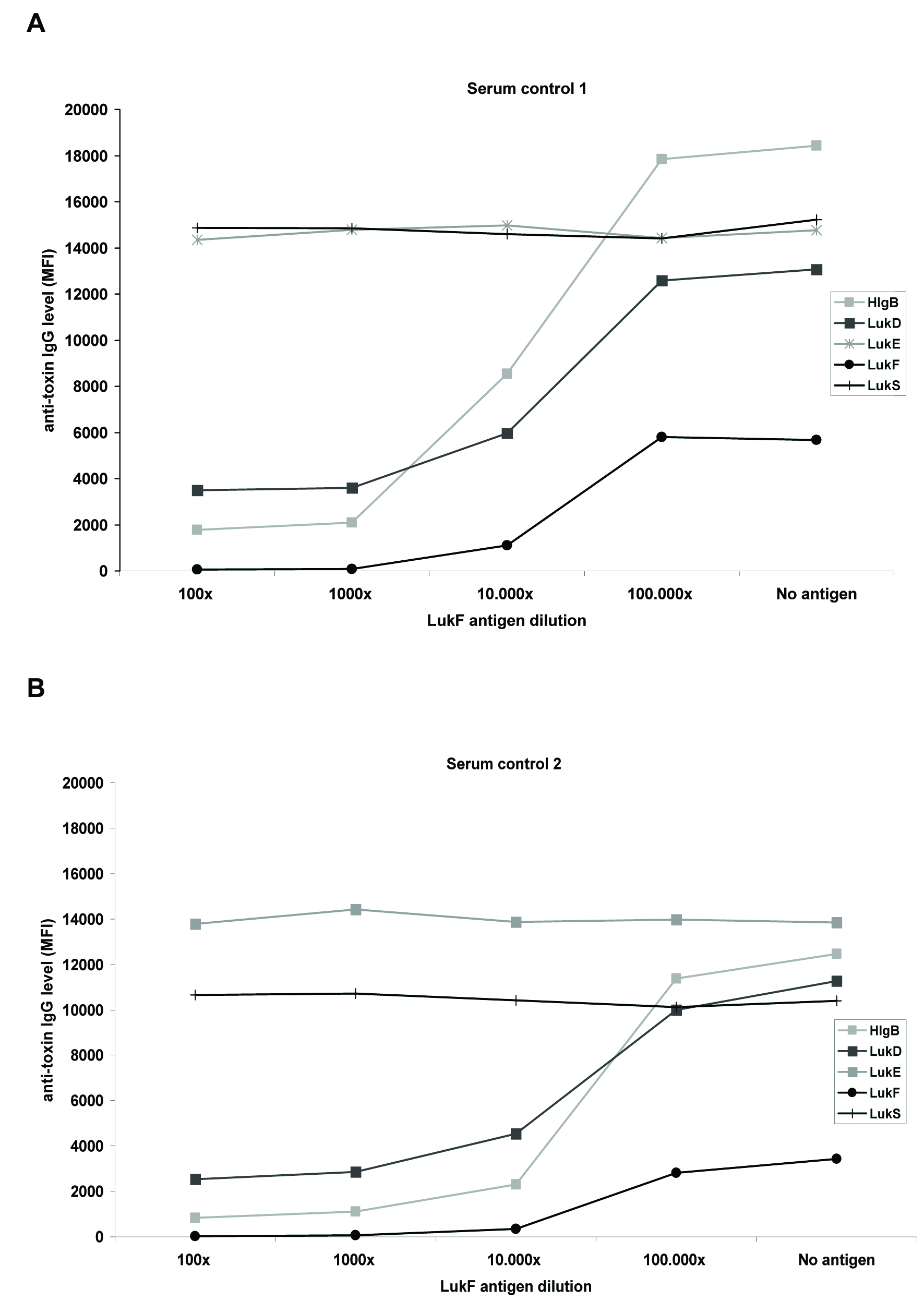

Supplement: Figure S1 — Cross-reactivity between leukocidins F and D and hemolysin gamma-B in human serum. A: Serial dilutions of recombinant leukocidin F (LukF) were pre-incubated with the serum from a non-infected control with high IgG levels against LukF. After incubation, the remaining IgG levels specific against Leukocidins D, E, F and S and Hemolysin gamma-B were measured. Note the loss in IgG levels specific for LukD and HlgB at lower dilutions of LukF, suggesting immunological cross-talk between these toxin components. B: The same experiment as for Figure S1 A, now with serum from a different non-infected control. (TIF) [file pone.0053391.s001.tif]
