# Supplementary material for: Characterization of the Humoral Immune Response during Staphylococcus aureus Bacteremia and Global Gene Expression by Staphylococcus aureus in Human Blood
Source: PLoS One. 2013 Jan 7;8(1):e53391. doi: 10.1371/journal.pone.0053391 (PMC3538780; doi:10.1371/journal.pone.0053391)
Supplement: Table S1 — Overview of gene presence and associated IgA responses of bacterial antigens. Presence of genes in 21 isolates and initial-to-peak fold-increases in IgA levels in 21 bacteremia patients for 56 staphylococcal antigens. Patients for whom the duplicate measurements of the IgA levels had a CV larger than 25% were excluded from the analysis. IgA levels for the antigens EsxA, EsxB, PSM alpha 1–4 peptides, SA0104, SEI, SEJ and SEO were completely excluded because of very low signal intensities with coefficients of variation larger than 25% for a majority of patients. ND: not determined. (DOC) [file pone.0053391.s002.doc]

| Antigen | No. of patients with gene pos isolates (%) | No of patients with increase in IgA level (%) | Median fold increase from initial to peak level (range |
| --- | --- | --- | --- |
| Alpha toxin | 21/21 (100%) | 17/21 (81%) | 1.22 (0.59-4.34) |
| CHIPS | 13/21 (62%) | 16/20 (80%) | 1.13 (0.52-4.67) |
| ClfA | 21/21 (100%) | 18/21 (86%) | 1.77 (0.37-19.18) |
| ClfB | 21/21 (100%) | 16/20 (80%) | 1.31 (0.22-17.9) |
| Efb | 20/21 (95%) | 6/6 (100%) | 1.28 (1.08-8.32) |
| EsxA | 21/21 (100%) | CV>25% | ND |
| EsxB | 14/21 (67%) | CV>25% | ND |
| ETA | 1/21 (5%) | 11/15 (73%) | 1.40 (0.41-5.33) |
| ETB | 0/21 (0%) | 17/21 (81%) | 1.38 (0.2-17.25) |
| FlipR | 15/21 (72%) | 12/14 (86%) | 1.64 (0.5-78.8) |
| FnbpA | 19/21 (91%) | 13/16 (81%) | 1.36 (0.25-19.11) |
| FnbpB | 6/21 (29%) | 12/16 (75%) | 1.42 (0.16-6.24) |
| Glucosaminidase | 21/21 (100%) | 18/20 (90%) | 1.69 (0.63-5.48) |
| HlgB | 16/21 (76%) | 15/21 (71%) | 1.27 (0.46-4.63) |
| IsaA | 21/21 (100%) | 18/20 (91%) | 1.39 (0.57-36.52) |
| IsdA | 21/21 (100%) | 18/21 (86%) | 1.92 (0.61-10.1) |
| IsdH | 20/21 (95%) | 18/19 (95%) | 2.03 (0.24-25.4) |
| Lipase | 21/21 (100%) | 17/21 (81%) | 1.56 (0.27-17.79) |
| LukD | 15/21 (71%) | 17/21 (81%) | 1.35 (0.67-10.32) |
| LukE | 15/21 (71%) | 18/21 (86%) | 1.40 (0.76-16.65) |
| LukF | 0/21 (0%) | 17/21 (81%) | 1.42 (0.15-5.16) |
| LukS | 0/21 (0%) | 16/20 (80%) | 1.35 (0.63-36.67) |
| LytM | 21/21 (100%) | 14/20 (70%) | 1.30 (0.17-19.11) |
| Nuc | 21/21 (100%) | 14/16 (88%) | 1.57 (0.11-16.63) |
| Peptidoglycan | ND | 6/8 (75%) | 1.29 (0.66-3.61) |
| PrsA | 21/21 (100%) | 5/5 (100%) | 3.92 (1.02-20.83) |
| PSMa peptides 1-4 | 21/21 (100%) | CV>25% | ND |
| SA0104 | 16/21 (76%) | CV>25% | ND |
| SA0486 | 17/21 (81%) | 11/13 (85%) | 1.4 (0.4-23.38) |
| SA0688 | 21/21 (100%) | 13/14 (93%) | 2.07 (0.67-11.31) |
| SasG | 11/21 (52%) | 14/15 (93%) | 2.51 (0.63-13.02) |
| SCIN | 20/21 (95%) | 19/21 (91%) | 1.43 (0.85-16.78) |
| SdrD | 17/21 (81%) | 16/17 (94%) | 1.85 (0.14-7.07) |
| SdrE | 14/21 (67%) | 14/15 (93%) | 1.46 (0.51-7.03) |
| SEA | 2/21 (14%) | 18/20 (91%) | 1.61 (0.46-8.69) |
| SEB | 5/21 (24%) | 13/17 (77%) | 1.32 (0.19-11.95) |
| SEC | 1/21 (5%) | 17/21 (81%) | 1.27 (0.53-13.82) |
| SED | 2/21 (10%) | 17/19 (90%) | 1.52 (0.45-3.87) |
| SEE | 0/21 (0%) | 11/15 (73%) | 1.30 (0.16-24.28) |
| SEG | 11/21 (52%) | 17/20 (85%) | 1.43 (0.12-3.34) |
| SEH | 0/21 (0%) | 14/17 (82%) | 1.60 (0.54-10.04) |
| SEI | 11/21 (52%) | CV>25% | ND |
| SEJ | 2/21 (10%) | CV>25% | ND |
| SEM | 8/21 (38%) | 15/17 (88%) | 1.55 (0.24-7.96) |
| SEN | 10/21 (48%) | 15/18 (83%) | 1.52 (0.23-10.09) |
| SEO | 11/21 (52%) | CV>25% | ND |
| SEQ | 1/21 (5%) | 8/12 (67%) | 1.32 (0.43-10.75) |
| SER | 2/21 (10%) | 13/17 (76%) | 1.46 (0.41-8.32) |
| SSL1 | 18/21 (86%) | 16/19 (84%) | 1.45 (0.63-5.38) |
| SSL3 | 19/21 (91%) | 15/21 (71%) | 1.42 (0.28-4.96) |
| SSL5 | 21/21 (100%) | 17/21 (81%) | 1.44 (0.59-21.96) |
| SSL9 | 6/21 (29%) | 16/20 (80%) | 1.52 (0.21-10.57) |
| SSL10 | 14/21 (67%) | 17/21 (81%) | 1.43 (0.53-6.29) |
| SSL11 | 7/21 (33%) | 15/19 (79%) | 1.54 (0.31-29.5) |
| TSST1 | 3/21 (14%) | 16/21 (76%) | 1.52 (0.58-8.97) |
| Wall teichoic acid | ND | 17/21 (81%) | 1.19 (4.67-0.52) |
