# Supplementary material for: Characterization of the Humoral Immune Response during Staphylococcus aureus Bacteremia and Global Gene Expression by Staphylococcus aureus in Human Blood
Source: PLoS One. 2013 Jan 7;8(1):e53391. doi: 10.1371/journal.pone.0053391 (PMC3538780; doi:10.1371/journal.pone.0053391)
Supplement: Table S2 — List of genes with altered mRNA expression in human blood. Genes are listed for which mRNA expression is respectively at least twofold increased or decreased in both isolates during all time points (30, 60 and 90 minutes) of culture in blood compared to the transcriptomes at the start of culture in blood (0 minutes). mRNA expression is quantified as the average RNA:DNA log ratio of duplo experiments in separate blood samples of two blood donors. Ranges of RNA:DNA log ratios between duplo experiments in separate blood samples are given, unless only a single measurement from one blood sample was available. (DOC) [file pone.0053391.s003.doc]

| At least twofold increase in mRNA expression | | Culture in blood |  |  |  |
| --- | --- | --- | --- | --- | --- |
| Gene | Log phase growth in BHI broth | 0 minutes | 30 minutes | 60 minutes | 90 minutes |
| SAR1524 | 6,726 (5,471 to 7,471) | 0,01 (0,01 to 0,0917)* | 5,525 | 36,02 | 0,86 |
| SACOL0045 | 1,699 (0,27 to 4,822) | 0,0674 | 2,298 | 0,398 | 200.012 |
| SA0743 | 4,05 (0,57 to 9,145) | 0,428 (0,253 to 0,602) | 12,26 (1,808 to 83,14) | 6,865 | 0,896 |
| SA1635 | 0,509 (0,192 to 2,403) | 0,0365 (0,01 to 0,133) | 0,655 | 0,278 (0,157 to 0,559) | 0,706 (0,12 to 2,03) |
| SAR1962 | 0,634 (0,356 to 1,13) | 0,118 | 1,147 (1,092 to 1,205) | 0,998 | 0,491 (0,406 to 0,594) |
| hlgA | 0,59 (0,178 to 56,8) | 0,273 (0,01 to 5,849) | 2,437 (0,272 to 12,41) | 4,118 (1,388 to 12,33) | 3,923 (1,463 to 10,68) |
| hlgB | 0,396 (0,102 to 15,73) | 0,166 (0,0105 to 2,615) | 1,165 (0,158 to 7,127) | 1,893 (0,601 to 6,751) | 2,824 (0,99 to 10,25) |
| SAOUHSC_02294 | 2,406 (1,416 to 3,905) | 1,096 | 7,05 (2,847 to 17,72) | 89,51 | 3,34 (1,398 to 7,979) |
| SAR1523 | 1,637 (0,563 to 11,93) | 1,144 (0,377 to 2,406) | 7,089 (6,25 to 8,041) | 5,18 (3,348 to 8,014) | 0,332 |
| uhpT | 0,39 (0,156 to 0,793) | 0,362 (0,0172 to 2,759) | 2,186 (0,318 to 6,009) | 4,085 (1,244 to 93,81) | 2,366 (1,186 to 5,309) |
| hlgC | 0,379 (0,138 to 14,55) | 0,179 (0,01 to 2,539) | 1,064 (0,179 to 3,859) | 1,81 (0,541 to 6,552) | 2,379 (0,765 to 9,058) |
| SAR0295 | 0,842 (0,712 to 0,969) | 0,0735 (0,01 to 0,722) | 0,403 (0,234 to 0,508) | 1,198 (0,139 to 19,2) | 0,489 (0,454 to 0,524) |
| SAOUHSC_00745 | 1,318 (0,414 to 4,042) | 0,134 (0,01 to 0,64) | 0,714 (0,331 to 1,451) | 0,627 (0,282 to 0,798) | 2,882 (0,224 to 23,51) |
| SAR1126 | 0,837 (0,429 to 1,86) | 0,343 (0,01 to 1,605) | 1,776 (0,919 to 9,079) | 1,433 (1,429 to 1,436) | 0,822 (0,73 to 0,926) |
| SACOL0642 | 0,539 (0,201 to 1,237) | 0,0816 (0,0384 to 0,166) | 0,411 (0,394 to 0,428) | 0,446 (0,329 to 0,801) | 0,921 (0,443 to 1,398) |
| MW0372 | 1,018 (0,243 to 29,77) | 0,129 (0,01 to 0,62) | 0,637 (0,282 to 3,156) | 0,499 (0,352 to 0,87) | 0,438 (0,01 to 1,001) |
| 8325B-0490 | 0,433 (0,148 to 1,12) | 0,101 (0,017 to 0,924) | 0,484 (0,236 to 1,016) | 0,439 (0,29 to 0,84) | 0,861 (0,485 to 1,237) |
| SAR2634 | 1,432 (0,566 to 7,618) | 0,787 (0,0275 to 3,978) | 3,244 (0,716 to 9,414) | 3,267 (1,39 to 5,291) | 5,176 (1,62 to 23,56) |
| MW0393v2 | 1,551 (0,394 to 103,4) | 0,176 (0,01 to 0,797) | 0,725 (0,272 to 2,018) | 0,653 (0,359 to 1,133) | 0,603 (0,468 to 0,82) |
| MW0369 | 0,767 (0,183 to 1,913) | 0,213 (0,01 to 0,783) | 0,776 (0,47 to 0,918) | 0,933 (0,67 to 1,448) | 1,685 (1,579 to 1,791) |
| gap2 | 0,924 (0,476 to 1,934) | 1,157 (0,216 to 9,865) | 4,188 (0,62 to 11,79) | 4,418 (0,984 to 10,7) | 121,4 (1,15 to 203.411) |
| SAR0222 | 0,693 (0,196 to 56,44) | 0,24 (0,0139 to 1,378) | 0,856 (0,393 to 3,059) | 0,852 (0,203 to 2,582) | 0,971 (0,409 to 2,974) |
| repA | 1,481 (0,21 to 4,744) | 0,16 | 0,562 (0,377 to 0,908) | 0,434 (0,142 to 1,329) | 0,769 (0,0802 to 2,562) |
| SAR2512 | 0,258 (0,0592 to 17,01) | 0,0959 (0,01 to 0,773) | 0,33 (0,0972 to 1,289) | 0,243 (0,01 to 0,993) | 0,524 (0,143 to 1,11) |
| SA2011 | 3,869 (0,422 to 36,76) | 0,938 | 3,174 (2,264 to 4,448) | 9,72 | 0,381 |
| SAR1338 | 1,233 (0,416 to 52,45) | 0,414 (0,01 to 1,229) | 1,306 (0,525 to 2,676) | 1,603 (0,968 to 2,9) | 2,1 (0,798 to 5,335) |
| SAR0210 | 0,51 (0,259 to 0,878) | 0,378 (0,0818 to 0,815) | 1,167 (0,193 to 3,149) | 1,779 (0,788 to 3,542) | 1,537 (0,852 to 4,786) |
| SAR0169 | 0,742 (0,24 to 1,544) | 0,877 (0,154 to 5,945) | 2,659 (0,215 to 7,705) | 2,388 (0,9 to 6,896) | 2,357 (0,993 to 5,555) |
| SAR1526 | 12,15 (2,651 to 53,26) | 0,569 (0,467 to 0,671) | 1,614 (1,316 to 1,794) | 13,78 (3,05 to 62,29) | 1,797 |
| SA0357 | 0,294 (0,107 to 0,753) | 0,129 (0,01 to 0,37) | 0,363 (0,199 to 0,472) | 0,363 (0,119 to 0,667) | 0,313 (0,201 to 0,746) |
| SAR0311 | 0,162 (0,108 to 0,245) | 0,179 (0,0321 to 0,668) | 0,498 (0,121 to 6,489) | 0,57 (0,328 to 1,596) | 0,657 (0,459 to 1,1) |
| SAR1849 | 1,27 (0,455 to 2,613) | 0,98 (0,01 to 2,974) | 2,706 (0,649 to 8,772) | 3,273 (1,605 to 8,594) | 2,803 (0,958 to 7,525) |
| lrgB | 0,764 (0,0584 to 4,818) | 1,276 (0,538 to 4,067) | 3,454 (0,646 to 18,86) | 4,489 (1,48 to 8,296) | 4,906 (3,815 to 7,804) |
| SAR0761 | 1,448 (0,504 to 5,323) | 2,165 (0,772 to 8,913) | 5,776 (1,083 to 12,05) | 8,367 (2,418 to 29,99) | 7,599 (3,119 to 32,95) |
| SAR0918 | 1,135 (0,433 to 3,061) | 0,524 (0,01 to 1,668) | 1,383 (0,692 to 4,595) | 1,281 (0,878 to 1,778) | 1,323 (0,7 to 2,364) |
| SAR0211 | 0,403 (0,167 to 0,693) | 0,382 (0,145 to 0,869) | 0,981 (0,229 to 3,496) | 1,77 (0,859 to 2,818) | 1,465 (0,791 to 2,769) |
| SAR0208 | 0,411 (0,235 to 0,722) | 0,361 (0,175 to 0,597) | 0,922 (0,141 to 2,544) | 1,482 (0,748 to 3,076) | 1,129 (0,525 to 2,148) |
| oppD | 0,353 (0,136 to 0,583) | 0,253 (0,0445 to 0,653) | 0,64 (0,151 to 1,934) | 0,752 (0,338 to 2,597) | 0,951 (0,535 to 1,877) |
| SAR2668 | 0,649 (0,281 to 5,646) | 0,317 (0,0268 to 0,754) | 0,799 (0,354 to 1,489) | 1,222 (0,844 to 2,522) | 1,356 (0,891 to 3,066) |
| SAR0719 | 0,547 (0,2 to 10,93) | 0,242 (0,01 to 0,662) | 0,608 (0,302 to 1,571) | 0,517 (0,27 to 0,924) | 0,731 (0,42 to 1,824) |
| hutI | 0,64 (0,226 to 40,76) | 0,152 (0,01 to 0,461) | 0,378 (0,144 to 0,919) | 0,626 (0,313 to 1,047) | 0,725 (0,462 to 0,975) |
| lrgA | 0,86 (0,244 to 3,329) | 1,309 (0,348 to 3,674) | 3,207 (0,35 to 14,07) | 4,861 (1,275 to 11,51) | 3,413 (1,389 to 7,175) |
| SAR0414a | 1,113 (0,405 to 3,114) | 0,219 (0,0655 to 0,728) | 0,533 (0,451 to 0,581) | 0,587 (0,109 to 1,064) | 0,634 (0,453 to 0,746) |
| SAR0996 | 1,523 (0,291 to 4,635) | 2,564 (0,646 to 6,639) | 6,165 (0,869 to 19,6) | 10,9 (1,792 to 75,96) | 7,25 (2,794 to 19,12) |
| SAR1143 | 0,802 (0,27 to 49,25) | 0,305 (0,01 to 2,07) | 0,732 (0,168 to 2,308) | 1,346 (0,55 to 4,128) | 2,437 (1,119 to 5,195) |
| SAR2414 | 0,815 (0,244 to 58,86) | 0,168 (0,01 to 0,499) | 0,4 (0,223 to 0,833) | 0,353 (0,21 to 1,094) | 0,43 (0,166 to 0,761) |
| COLB3543 | 0,859 (0,188 to 90,35) | 0,113 (0,01 to 0,298) | 0,268 (0,109 to 1,316) | 0,357 (0,131 to 0,808) | 0,585 (0,432 to 0,856) |
| SAR0206 | 0,459 (0,265 to 0,905) | 0,374 (0,184 to 0,785) | 0,883 (0,142 to 4,506) | 0,846 (0,495 to 1,238) | 0,805 (0,453 to 1,613) |
| fadA | 0,444 (0,125 to 30,61) | 0,108 (0,01 to 0,433) | 0,253 (0,0975 to 0,739) | 0,292 (0,137 to 0,5) | 0,426 (0,117 to 0,606) |
| SAP008 | 1,226 (0,425 to 40) | 0,313 (0,01 to 1,217) | 0,734 (0,504 to 1,128) | 1,007 (0,528 to 2,324) | 0,744 (0,587 to 1,014) |
| hlb | 0,506 (0,233 to 0,83) | 0,495 (0,178 to 3,262) | 1,161 (0,364 to 3,347) | 1,699 (0,722 to 3,853) | 1,941 (1,058 to 3,578) |
| SAR0308 | 0,195 (0,0911 to 0,502) | 0,158 (0,0384 to 1,41) | 0,369 (0,13 to 1,987) | 0,487 (0,197 to 2,126) | 1,134 (0,363 to 3,512) |
| rocD | 1,109 (0,517 to 2,064) | 1,141 (0,589 to 2,577) | 2,65 (0,822 to 8,286) | 2,619 (1,38 to 5,197) | 2,884 (1,104 to 7,396) |
| fadX | 0,295 (0,0959 to 14,02) | 0,096 (0,01 to 0,372) | 0,223 (0,0709 to 0,705) | 0,264 (0,0803 to 1,035) | 0,453 (0,241 to 0,737) |
| hutU | 0,43 (0,155 to 9,417) | 0,133 (0,01 to 0,501) | 0,303 (0,0898 to 0,873) | 0,461 (0,257 to 0,734) | 0,692 (0,351 to 1,413) |
| SAOUHSC_02990 | 1,945 (0,107 to 28.150) | 0,0655 (0,01 to 0,255) | 0,149 (0,0791 to 0,195) | 0,287 (0,121 to 0,506) | 0,208 (0,13 to 0,377) |
| SAR2641 | 2,105 (0,242 to 60.139) | 0,235 (0,01 to 0,702) | 0,532 (0,239 to 1,033) | 0,622 (0,361 to 1,075) | 0,713 (0,467 to 1,433) |
| SAR2616 | 0,747 (0,158 to 1,37) | 0,448 (0,01 to 1,129) | 1,011 (0,653 to 1,372) | 1,192 (0,721 to 2,025) | 1,541 (1,227 to 2,314) |
| SAR0119 | 0,188 (0,0847 to 0,556) | 0,162 (0,01 to 1,429) | 0,366 (0,127 to 0,755) | 0,479 (0,213 to 1,468) | 1,372 (0,622 to 3,992) |
| SAR1987 | 1,274 (0,662 to 1,879) | 0,41 | 0,921 (0,561 to 1,557) | 0,831 (0,759 to 0,902) | 0,924 (0,875 to 0,975) |
| fadD | 0,488 (0,103 to 37,96) | 0,0799 (0,01 to 0,389) | 0,18 (0,0534 to 0,517) | 0,234 (0,114 to 0,645) | 0,394 (0,272 to 0,497) |
| SAR0217 | 5,957 (0,01 to 28,42) | 6,679 (0,231 to 28,36) | 14,83 (6,321 to 26,72) | 24,25 (14,73 to 55,8) | 20,73 (10,55 to 53,49) |
| asd | 0,406 (0,0974 to 8,158) | 0,164 (0,01 to 0,872) | 0,365 (0,153 to 0,738) | 0,455 (0,125 to 3,409) | 0,514 (0,212 to 0,908) |
| SAR2513 | 0,429 (0,0925 to 136,1) | 0,112 (0,01 to 0,595) | 0,248 (0,049 to 0,975) | 0,287 (0,155 to 0,651) | 0,422 (0,193 to 1,197) |
| pyrC | 1,288 (0,285 to 3,188) | 1,222 (0,52 to 2,741) | 2,703 (0,995 to 7,421) | 2,83 (1,056 to 6,317) | 2,894 (2,517 to 3,867) |
| thrC | 0,982 (0,453 to 22,15) | 0,366 (0,01 to 1,075) | 0,808 (0,283 to 1,432) | 0,966 (0,495 to 1,842) | 1,241 (0,679 to 2,936) |
| SAR2580v | 1,085 (0,18 to 3,81) | 0,551 (0,01 to 3,511) | 1,214 (0,389 to 2,381) | 1,447 (0,325 to 6,238) | 1,577 (0,452 to 4,953) |
| SAR0660 | 0,77 (0,347 to 1,615) | 0,832 (0,412 to 1,806) | 1,822 (0,601 to 2,98) | 1,831 (0,91 to 3,766) | 1,71 (1,162 to 2,541) |
| SAR0312 | 0,372 (0,149 to 7,184) | 0,172 (0,0156 to 0,727) | 0,376 (0,175 to 1,263) | 0,57 (0,287 to 0,846) | 0,728 (0,458 to 1,57) |
| fmtB | 0,439 (0,0946 to 22,45) | 0,152 (0,01 to 0,76) | 0,333 (0,134 to 0,667) | 0,445 (0,135 to 1,265) | 0,632 (0,449 to 0,995) |
| SAR0129 | 1,306 (0,393 to 3,207) | 1,902 (0,343 to 5,725) | 4,135 (0,615 to 7,559) | 6,704 (2,696 to 32,19) | 5,406 (2,419 to 8,16) |
| SAR2104 | 0,658 (0,317 to 3,064) | 0,475 (0,106 to 0,772) | 1,03 (0,495 to 3,354) | 1,536 (0,748 to 3,379) | 1,051 (0,828 to 1,402) |
| SACOL0323 | 0,917 (0,144 to 13,06) | 0,162 (0,01 to 0,388) | 0,351 (0,147 to 0,811) | 0,342 (0,156 to 0,518) | 0,494 (0,459 to 0,579) |
| bioA | 0,246 (0,0708 to 8,204) | 0,0711 (0,01 to 0,233) | 0,152 (0,0518 to 0,708) | 0,151 (0,01 to 0,337) | 0,249 (0,0679 to 0,465) |
| COLB0874 | 0,779 (0,15 to 34,05) | 0,107 (0,01 to 0,274) | 0,227 (0,121 to 0,389) | 0,361 (0,01 to 0,753) | 0,431 (0,207 to 0,992) |
| SAR0209 | 0,591 (0,364 to 1,164) | 0,619 (0,182 to 1,616) | 1,319 (0,195 to 5,91) | 1,883 (0,671 to 3,155) | 1,453 (0,605 to 2,572) |
| thrB | 1,181 (0,418 to 70,58) | 0,394 (0,01 to 1,066) | 0,835 (0,421 to 1,436) | 0,827 (0,49 to 1,563) | 0,978 (0,657 to 1,432) |
| sbi | 2,559 (0,804 to 159,6) | 2,029 (0,963 to 6,119) | 4,296 (2,15 to 7,031) | 6,016 (3,985 to 14,87) | 4,922 (2,199 to 14,45) |
| SAR0316 | 0,427 (0,134 to 5,212) | 0,124 (0,01 to 0,39) | 0,262 (0,105 to 0,764) | 0,268 (0,107 to 0,69) | 0,423 (0,01 to 0,872) |
| SAR0200 | 0,229 (0,0932 to 0,457) | 0,124 (0,01 to 0,617) | 0,261 (0,161 to 0,496) | 0,401 (0,133 to 1,094) | 1,151 (0,968 to 1,334) |
| sirA | 0,664 (0,249 to 2,662) | 1,189 (0,297 to 4,779) | 2,489 (0,371 to 5,396) | 3,179 (0,823 to 8,7) | 5,136 (1,91 to 11,77) |
| SA1789 | 0,668 (0,134 to 118,8) | 0,0624 (0,01 to 0,109) | 0,13 (0,0735 to 0,21) | 0,236 (0,01 to 9,812) | 0,481 (0,423 to 0,654) |
| SAR0207 | 0,393 (0,2 to 0,649) | 0,355 (0,128 to 0,81) | 0,738 (0,157 to 2,501) | 1,193 (0,558 to 2,001) | 1,001 (0,547 to 1,81) |
| isdC | 0,26 (0,0921 to 0,53) | 0,34 (0,0888 to 3,518) | 0,691 (0,174 to 2,296) | 1,041 (0,428 to 3,383) | 2,798 (1,519 to 6,09) |
| oppF | 0,244 (0,108 to 0,517) | 0,274 (0,0997 to 0,753) | 0,556 (0,199 to 1,454) | 0,739 (0,365 to 1,953) | 0,944 (0,483 to 2,111) |
| SAR0760 | 1,425 (0,536 to 3,851) | 1,531 (0,751 to 3,011) | 3,08 (1,527 to 5,218) | 3,132 (1,634 to 7,887) | 3,118 (1,105 to 11,93) |
|  |  |  |  |  |  |
| At least twofold decrease in mRNA expression | | Culture in blood |  |  |  |
| Gene | Log phase growth in BHI broth | 0 minutes | 30 minutes | 60 minutes | 90 minutes |
| fadB | 0,378 (0,127 to 3,842) | 0,0954 (0,01 to 0,545) | 0,192 (0,0307 to 0,64) | 0,449 (0,206 to 2,577) | 0,44 (0,192 to 0,946) |
| sdrC | 2,594 (0,246 to 8,085) | 8,388 (6,535 to 10,89) | 4,128 (2,567 to 6,087) | 3,211 (2,223 to 5,509) | 2,001 (1,699 to 2,431) |
| SAR0972 | 2,598 (2,567 to 2,629) | 3,57 | 1,7 (0,98 to 3,086) | 1,087 (1,047 to 1,126) | 1,023 |
| SAR2127 | 0,86 (0,405 to 1,744) | 1,75 (0,421 to 300,1) | 0,777 (0,516 to 1,492) | 0,874 (0,456 to 2,121) | 0,741 (0,201 to 1,249) |
| cspB | 2,904 (0,98 to 5,615) | 4,732 (1,9 to 10,09) | 2,034 (1,057 to 6,669) | 2,071 (1,326 to 2,674) | 1,914 (0,858 to 2,694) |
| uvrC | 2,283 (1,939 to 2,626) | 1,768 (1,526 to 2,011) | 0,722 (0,612 to 0,879) | 0,862 (0,558 to 1,662) | 0,87 (0,574 to 1,461) |
| SAR0458 | 0,841 (0,433 to 2,085) | 1,426 (0,919 to 2,157) | 0,555 (0,265 to 1,095) | 0,522 (0,234 to 1,096) | 0,395 (0,257 to 0,767) |
| SAR1008 | 4,736 (1,673 to 12,24) | 2,597 (2,525 to 2,668) | 0,997 (0,83 to 1,209) | 0,964 (0,569 to 1,928) | 0,724 (0,658 to 0,824) |
| COLB2006 | 1,715 (0,127 to 41,13) | 3,363 (0,949 to 76,22) | 1,267 (0,798 to 2,629) | 1,312 (0,893 to 1,672) | 0,746 (0,517 to 1,141) |
| SAOUHSC_02223 | 1,519 (1,047 to 2,93) | 11 (0,993 to 121,7) | 4,127 (2,411 to 7,065) | 1,976 | 0,772 |
| COLB3502 | 6,534 (1,966 to 156,4) | 15,69 (1,477 to 84.610) | 5,881 (2,704 to 12,83) | 41,2 (3,236 to 20.024) | 2,737 (1,498 to 3,513) |
| SAR0905 | 2,497 (0,662 to 16,49) | 2,301 (0,648 to 9,762) | 0,83 (0,497 to 2,823) | 0,775 (0,353 to 2,209) | 0,642 (0,351 to 1,226) |
| SAR1238 | 10,03 (0,492 to 273,3) | 2,647 (1,732 to 3,983) | 0,93 (0,81 to 1,086) | 0,956 (0,69 to 1,324) | 1,309 (1,186 to 1,604) |
| SAR0460 | 1,539 (0,918 to 2,864) | 1,448 (1,374 to 1,521) | 0,485 (0,278 to 0,818) | 0,69 (0,428 to 1,241) | 0,585 (0,324 to 1,087) |
| dltD | 5,316 (1,539 to 14,61) | 5,071 (1,075 to 22,33) | 1,655 (0,875 to 4,597) | 1,334 (0,761 to 2,447) | 1,294 (0,906 to 2,187) |
| SAR2049 | 0,93 (0,495 to 1,767) | 0,91 | 0,247 (0,123 to 0,654) | 0,327 (0,256 to 0,397) | 0,41 (0,248 to 0,764) |
| fhuA | 0,874 (0,416 to 2,522) | 4,626 (0,338 to 229.580) | 1,227 (0,649 to 2,116) | 0,999 (0,62 to 1,476) | 1,212 (0,824 to 1,817) |
| tsf | 7,023 (1,672 to 24,29) | 33,13 (4,231 to 95.058) | 8,635 (5,317 to 13,46) | 8,334 (3,364 to 18,18) | 8,638 (3,369 to 21,99) |
| rpsD | 5,996 (2,868 to 17,56) | 31,47 (5,429 to 188.403) | 7,828 (3,856 to 18,27) | 6,648 (2,417 to 21,32) | 8,495 (5,273 to 15,47) |
| SAB0725 | 3,577 (0,712 to 24,23) | 50,08 (3,009 to 667.968) | 12,23 (4,834 to 51,48) | 19,6 (4,994 to 56,22) | 103,5 (2,664 to 223.501) |
| SAR1059 | 2,32 (1,246 to 6,465) | 8,598 (0,683 to 96.257) | 1,994 (0,927 to 3,852) | 1,698 (0,712 to 3,53) | 1,796 (1,266 to 2,253) |
| atpD | 10,6 (3,214 to 17,96) | 33,66 (3,131 to 83.754) | 7,792 (0,948 to 20,46) | 6,696 (1,927 to 25,79) | 7,25 (0,959 to 26,59) |
| rpmD | 36,53 (12,99 to 145,9) | 131,4 (23,33 to 417.399) | 28,73 (13,48 to 77) | 30,75 (8,603 to 120,2) | 26,67 (7,914 to 71,95) |
| SAOUHSC_A01455 | 1,28 (0,887 to 2,292) | 19,64 (1,053 to 113.727) | 3,386 (1,3 to 8,417) | 3,282 (1,628 to 7,026) | 0,7 |
| SAR2024 | 2,704 (0,331 to 30,26) | 4,297 (1,149 to 16,07) | 0,664 (0,543 to 0,874) | 0,925 (0,861 to 0,989) | 0,73 |
| SAR0297 | 0,579 (0,369 to 0,793) | 7,384 (0,408 to 242.415) | 0,601 (0,15 to 2,22) | 0,26 (0,211 to 0,399) | 0,352 (0,307 to 0,473) |
| tnpA2 | 0,396 (0,306 to 0,517) | 7,307 (0,231 to 832.972) | 0,401 (0,199 to 1,244) | 0,422 (0,0905 to 1,685) | 0,358 (0,262 to 0,525) |
| SAR0473 | 2,355 (1,298 to 5,564) | 34,05 (2,402 to 88.369) | 1,809 (1,139 to 3,005) | 5,623 (3,873 to 7,839) | 2,257 (1,037 to 3,231) |
| SAR1894 | 1,78 (0,177 to 24,72) | 10,06 (0,243 to 117.324) | 0,313 (0,217 to 0,41) | 0,294 (0,118 to 0,517) | 0,403 (0,114 to 0,686) |
| dat | 5,294 (1,049 to 119) | 3.147 (1,751 to 164.598) | 2,628 (2,137 to 3,352) | 3,725 (2,861 to 4,296) | 3,106 (2,138 to 4,598) |
