# Supplementary material for: Characterization of the Humoral Immune Response during Staphylococcus aureus Bacteremia and Global Gene Expression by Staphylococcus aureus in Human Blood
Source: PLoS One. 2013 Jan 7;8(1):e53391. doi: 10.1371/journal.pone.0053391 (PMC3538780; doi:10.1371/journal.pone.0053391)
Supplement: Table S3 — mRNA expression levels of 35 genes in two isolates during culture in human blood and log-phase growth in BHI broth. Average RNA:DNA log ratios of duplicate experiments in two separate blood samples are given; dark (red) cells indicate a RNA:DNA ratio larger than 2 (i.e. high expression) and gray (blue) cell indicates a RNA:DNA ratio smaller than 0.5 (i.e. low expression). Range of RNA:DNA log ratios between duplo experiments in seperate blood samples are given, unless only a single measurement from one blood sample was available. (DOC) [file pone.0053391.s004.doc]

|  | **Isolate 1** | **Culture in blood** |  |  |  |
| --- | --- | --- | --- | --- | --- |
| Gene | **log phase growth in BHI broth** | **0 min** | **30 min** | **60 min** | **90 min** |
| **Luk F** | 0,41 (0,269 to 0,593) | 0,489 (0,276 to 0,75) | 0,529 (0,333 to 0,808) | 1,414 (0,815 to 2,38) | 1,573 (0,811 to 2,186) |
| **SEC** | 0,551 (0,328 to 1,053) | 0,311 (0,174 to 0,444) | 0,381 (0,207 to 0,639) | 0,433 (0,176 to 0,937) | 0,495 (0,374 to 0,686) |
| **SEE** | 6,292 |  | 4,52 |  |  |
| **FnbpA** | 0,659 (0,376 to 1,338) | 0,344 (0,01 to 1,424) | 1,247 (0,721 to 2,25) | 0,985 (0,544 to 1,486) | 0,949 (0,828 to 1,035) |
| **FnbpB** | 0,749 (0,495 to 1,192) | 0,815 (0,615 to 1,14) | 1,202 (0,437 to 2,115) | 0,777 (0,379 to 1,282) |  |
| **SEG** | 0,31 (0,227 to 0,375) | 0,329 (0,233 to 0,388) | 0,396 (0,278 to 0,594) | 0,291 (0,15 to 0,634) | 0,299 (0,0967 to 0,584) |
| **SEH** | 6,238 (1,15 to 33,84) | 2,496 | 1,439 (1,374 to 1,504) |  |  |
| **SdrD** | 6,652 (4,326 to 8,085) | 8,388 (6,535 to 10,89) | 4,595 (2,567 to 6,087) | 3,211 (2,223 to 5,509) | 2,001 (1,699 to 2,431) |
| **SEI** | 0,302 (0,125 to 0,45) | 0,269 (0,189 to 0,423) | 0,302 (0,207 to 0,352) | 0,251 (0,12 to 0,435) | 0,238 (0,0992 to 0,543) |
| **SEM** | 0,818 (0,339 to 3,195) | 0,671 (0,589 to 0,713) | 0,662 (0,479 to 0,833) | 0,889 (0,688 to 1,85) | 1,884 (0,734 to 3,034) |
| **SEN** | 0,48 (0,208 to 0,941) | 0,436 (0,333 to 0,49) | 0,368 (0,295 to 0,467) | 0,45 (0,298 to 0,624) | 1,533 (1,508 to 1,558) |
| **SdrE** | 1,586 (1,504 to 1,672) | 13,46 | 3,868 (2,514 to 5,221) |  |  |
| **ETA** | 4,123 (0,831 to 38,44) |  | 0,804 | 83,84 |  |
| **ClfA** | 1,104 (0,652 to 1,442) | 1,452 (0,753 to 2,283) | 2,013 (1,215 to 4,546) | 3,438 (2,8 to 4,156) | 2,226 (2,135 to 2,317) |
| **HlgB** | 0,218 (0,151 to 0,308) | 0,0707 (0,0105 to 0,187) | 1,303 (0,442 to 3,442) | 2,581 (1,274 to 6,751) | 2,019 (1,644 to 2,903) |
| **SCIN** | 3,055 (2,461 to 4,675) | 3,409 (2,048 to 5,067) | 6,59 (4,503 to 9,112) | 10,09 (4,311 to 23,9) | 7,915 (5,992 to 9,978) |
| **ClfB** | 1,141 (0,861 to 1,613) | 1,427 (1,036 to 1,92) | 1,274 (0,631 to 2,162) | 1,285 (0,857 to 2,241) | 1,381 (0,991 to 1,836) |
| **CHIPS** | 2,79 (0,768 to 7,76) | 0,908 | 0,5 (0,483 to 0,518) |  |  |
| **SasG** | 7,411 (1,627 to 35,52) |  | 2,987 |  |  |
| **IsdA** | 0,684 (0,172 to 3,296) | 0,475 | 0,734 (0,5 to 1,107) | 2,687 (1,582 to 4,119) |  |
| **Efb** | 1,907 (1,038 to 3,237) | 1,799 (0,738 to 3,319) | 5,355 (2,068 to 14,6) | 5,27 (3,921 to 7,864) | 2,947 |
| **Alpha toxin** | 1,685 (0,377 to 6,354) |  | 0,624 (0,577 to 0,671) |  |  |
| **Luk S** | 1,806 (1,372 to 2,398) | 0,48 (0,278 to 0,948) | 0,984 (0,692 to 1,399) | 0,715 |  |
| **SEO** | 0,404 (0,316 to 0,601) | 0,45 (0,161 to 0,784) | 0,191 (0,0273 to 0,681) | 0,229 (0,121 to 0,269) | 1,067 (0,799 to 1,335) |
| **SSL 1** | 0,233 (0,161 to 0,404) | 0,185 (0,102 to 0,355) | 0,282 (0,213 to 0,419) | 0,413 (0,292 to 0,535) | 0,446 (0,318 to 0,709) |
| **SSL 11** | 2,372 (1,583 to 3,161) |  | 0,889 |  |  |
| **FlipR** | 0,627 (0,453 to 0,74) | 0,689 (0,616 to 0,762) | 1,118 (0,832 to 1,405) | 2,097 (1,902 to 2,412) |  |
| **PrsA** | 5,467 (4,4 to 9,422) | 8,276 (7,211 to 10,6) | 5,117 (1,756 to 11,65) | 6,005 (4,759 to 8,495) | 5,275 (3,661 to 7,939) |
| **EsxB** | 0,309 (0,256 to 0,335) | 0,303 (0,161 to 0,953) | 0,293 (0,203 to 0,388) | 0,384 (0,179 to 0,737) | 0,334 (0,0997 to 0,594) |
| **EsxA** | 3,464 (1,14 to 6,933) | 4,048 (1,793 to 8,737) | 4,764 (2,811 to 9,135) | 10,74 (4,461 to 24,58) | 6,148 (2,764 to 14,98) |
| **IsaA** | 6,698 (4,752 to 11,17) | 10,43 (7,928 to 19,71) | 7,089 (4,009 to 10,3) | 7,179 (2,428 to 12,68) | 3,928 (3,263 to 4,593) |
| **SA0486** | 0,324 (0,0804 to 0,787) | 0,354 (0,167 to 1,821) | 0,488 (0,276 to 0,812) | 0,475 (0,286 to 0,675) | 0,395 (0,0533 to 0,547) |
| **SA0688** | 6,522 (3,927 to 11,53) | 18,15 (11,86 to 43,04) | 14,24 (8,091 to 21,98) | 12,02 (8,611 to 18,75) | 9,868 (6,401 to 17,13) |
| **lytM** | 0,629 (0,363 to 1,171) | 0,369 (0,0457 to 1,536) | 1,874 (1,343 to 2,408) | 0,708 (0,425 to 1,603) | 0,736 (0,57 to 0,998) |
| **Nuc** | 0,552 (0,466 to 0,686) | 0,269 (0,01 to 1,057) | 0,607 (0,28 to 1,033) | 0,654 (0,467 to 1,223) | 0,574 (0,234 to 0,758) |
|  |  |  |  |  |  |
|  | **Isolate 2** | **Culture in blood** |  |  |  |
|  | **log phase growth in BHI broth** | **0 min** | **30 min** | **60 min** | **90 min** |
| **Luk F** | 0,572 (0,235 to 2,123) | 0,945 (0,553 to 1,659) | 1,314 (0,104 to 2,95) | 1,236 (0,656 to 2,192) | 1,983 (0,852 to 4,115) |
| **SEC** | 0,349 (0,184 to 1,31) | 0,443 (0,212 to 1,027) | 0,282 (0,108 to 0,742) | 0,267 (0,175 to 0,39) | 0,265 (0,0609 to 0,717) |
| **SEE** | 0,847 (0,479 to 1,214) |  | 2,705 |  |  |
| **FnbpA** | 4,355 (2,51 to 17,82) | 2,112 (1,208 to 4,127) | 2,086 (1,116 to 3,542) | 3,743 (1,44 to 7,45) | 3,228 (2,164 to 5,152) |
| **FnbpB** | 0,801 (0,46 to 1,506) |  | 4,868 (3,074 to 7,709) |  | 0,299 |
| **SEG** | 0,383 (0,163 to 1,264) | 0,445 (0,211 to 1,391) | 0,309 (0,104 to 0,47) | 0,306 (0,155 to 0,555) | 0,299 (0,074 to 0,603) |
| **SEH** | 1,877 (1,767 to 1,987) | 3,005 | 3,539 (1,726 to 6,261) | 3,39 (2,823 to 3,808) | 2,065 (1,405 to 3,017) |
| **SdrD** | 0,739 (0,246 to 1,434) |  | 2,687 |  |  |
| **SEI** | 0,289 (0,18 to 0,89) | 0,392 (0,212 to 0,587) | 0,337 (0,263 to 0,503) | 0,243 (0,133 to 0,639) | 0,285 (0,0681 to 0,76) |
| **SEM** | 0,544 (0,307 to 1,4) | 0,624 (0,431 to 1,17) | 0,489 (0,408 to 0,625) | 0,374 (0,221 to 0,693) | 0,126 (0,0974 to 0,154) |
| **SEN** | 0,298 (0,205 to 0,523) | 0,452 (0,36 to 0,825) | 0,592 (0,478 to 0,91) | 0,411 (0,302 to 0,52) | 0,192 |
| **SdrE** | 1,492 (0,204 to 7,522) | 4,945 |  |  | 0,669 |
| **ETA** | 1,667 (0,948 to 2,932) |  | 1,366 |  | 0,583 |
| **ClfA** | 1,389 (0,604 to 8,518) | 0,819 (0,479 to 1,227) | 1,02 (0,624 to 2,407) | 1,388 (0,743 to 2,596) | 2,333 (1,436 to 3,458) |
| **HlgB** | 0,72 (0,102 to 15,73) | 0,392 (0,131 to 2,615) | 1,041 (0,158 to 7,127) | 1,389 (0,601 to 2,211) | 3,533 (0,99 to 10,25) |
| **SCIN** | 4,496 (2,576 to 8,511) | 7,389 (3,011 to 13,79) | 5,935 (2,879 to 11,53) | 4,952 (2,075 to 10,8) | 9,611 (5,103 to 14,12) |
| **ClfB** | 2,656 (1,459 to 8,001) | 2,074 (1,031 to 6,653) | 2,021 (1,078 to 3,827) | 3,307 (1,445 to 5,849) | 2,477 (1,487 to 4,681) |
| **CHIPS** | 2,229 (0,839 to 6,71) | 0,382 | 1,166 (0,393 to 2,115) | 0,421 (0,358 to 0,483) | 1,284 |
| **SasG** | 2,153 (0,312 to 14,97) |  |  |  | 0,251 (0,24 to 0,263) |
| **IsdA** | 0,662 (0,299 to 1,663) | 1,08 (0,265 to 5,326) | 1,379 (0,3 to 3,318) | 1,704 (0,546 to 4,601) | 3,238 (1,098 to 7,014) |
| **Efb** | 2,512 (0,967 to 6,788) | 2,701 (0,804 to 5,888) | 2,694 (0,709 to 6,002) | 2,888 (1,403 to 4,067) | 4,691 (2,055 to 7,58) |
| **Alpha toxin** | 3,034 (1,148 to 19,17) | 1,137 (0,794 to 1,481) | 0,847 (0,442 to 1,639) | 0,954 (0,596 to 1,876) | 0,671 (0,43 to 0,944) |
| **Luk S** | 0,526 (0,332 to 0,72) | 0,524 |  |  |  |
| **SEO** | 0,397 (0,124 to 1,253) | 0,506 (0,115 to 1,413) | 0,547 (0,285 to 1,046) | 0,366 (0,254 to 0,57) | 0,293 (0,01 to 0,823) |
| **SSL 1** | 1,319 (0,551 to 2,498) |  |  |  | 0,461 (0,436 to 0,486) |
| **SSL 11** | 1,343 (0,681 to 2,79) | 2,387 (2,204 to 2,571) | 1,782 (1,583 to 2,1) | 2,887 (1,341 to 4,742) | 2,642 (0,186 to 22,58) |
| **FlipR** | 0,678 (0,398 to 2,052) | 2,678 (0,98 to 7,59) | 1,85 (0,45 to 5,153) | 1,442 (0,897 to 2,323) | 2,453 (2,406 to 2,5) |
| **PrsA** | 1,887 (1,051 to 5,443) | 3,718 (2,512 to 6,346) | 2,927 (1,561 to 4,533) | 2,53 (1,519 to 4,665) | 1,856 (0,921 to 3,807) |
| **EsxB** | 0,686 (0,386 to 0,987) |  | 3,38 |  | 1,469 |
| **EsxA** | 2,374 (1,955 to 3,879) | 3,057 (1,241 to 6,928) | 3,099 (1,551 to 4,395) | 3,99 (2,311 to 8,532) | 5,093 (3,429 to 8,175) |
| **IsaA** | 8,747 (4,953 to 17,52) | 11,68 (6,206 to 34,71) | 7,667 (3,484 to 12,99) | 8,101 (3,39 to 27,13) | 8,322 (5,35 to 15,06) |
| **SA0486** | 0,226 (0,0902 to 0,677) | 0,268 (0,128 to 0,477) | 0,304 (0,139 to 0,535) | 0,287 (0,17 to 0,507) | 0,368 (0,148 to 0,627) |
| **SA0688** | 14,75 (0,215 to 253,6) | 7,648 (2,758 to 14,92) | 5,64 (0,972 to 9,216) | 7,11 (4,429 to 15,28) | 7,364 (5,102 to 12,75) |
| **lytM** | 1,397 (0,443 to 3,126) | 1,17 (0,243 to 2,867) | 1,413 (0,962 to 2,004) | 1,493 (0,543 to 2,519) | 1,875 (1,453 to 2,531) |
| **Nuc** | 3,39 (1,028 to 58,47) | 0,98 (0,6 to 1,826) | 0,834 (0,507 to 1,074) | 0,889 (0,623 to 1,445) | 0,81 (0,639 to 1,25) |
